# An International Clinical Study of Ability and Disability in Autism Spectrum Disorder Using the WHO-ICF Framework

**DOI:** 10.1007/s10803-018-3482-4

**Published:** 2018-02-08

**Authors:** Soheil Mahdi, Katja Albertowski, Omar Almodayfer, Vaia Arsenopoulou, Sara Carucci, José Carlos Dias, Mohammad Khalil, Ane Knüppel, Anika Langmann, Marlene Briciet Lauritsen, Graccielle Rodrigues da Cunha, Tokio Uchiyama, Nicole Wolff, Melissa Selb, Mats Granlund, Petrus J. de Vries, Lonnie Zwaigenbaum, Sven Bölte

**Affiliations:** 1 Center of Neurodevelopmental Disorders at Karolinska Institutet (KIND), Division of Neuropsychiatry, Department of Women’s and Children’s Health, Karolinska Institutet, 113 30 Stockholm, Sweden; 2 Center for Psychiatry Research, Stockholm County Council, Stockholm, Sweden; 3 Experimental Developmental Psychopathology, Department of Child and Adolescent Psychiatry, Faculty of Medicine of the TU Dresden, Dresden, Germany; 4 Mental Health Department, KAMC-R, MNGHA, Riyadh, Saudi Arabia; 5 Theotokos Foundation, Athens, Greece; 6 Child & Adolescent Neuropsychiatric Unit, Department of Biomedical Science, University of Cagliari & “A. Cao” Microcitemico Paediatric Hospital, Cagliari, Italy; 7 Childhood and Adolescence Psychiatry Department, Oporto Hospital Centre, Porto, Portugal; 8 Human Development Center, Riyadh, Saudi Arabia; 9 Aalborg University Hospital Psychiatry, Aalborg, Denmark; 10 Department of Child and Adolescent Psychiatry, Psychosomatics and Psychotherapy, University Hospital of Marburg & Institute of Clinical Psychology, Philipps-University Marburg, Marburg, Germany; 11 Aalborg University Hospital, Clinical Institute, Aalborg, Denmark; 12 TEAMM Clinic, Department of Psychiatry, Federal University of São Paulo (UNIFESP), São Paulo, Brazil; 13 Japan Centre for Applied Autism Research, Department of Clinical Psychology, Taisho University, Tokyo, Japan; 14 ICF Research Branch, A Cooperation Partner Within the WHO Collaborating Centre for the Family of International Classifications in Germany (at DIMDI), Nottwil, Switzerland; 15 Swiss Paraplegic Research, Nottwil, Switzerland; 16 CHILD, SIDR, School of Health and Welfare, Jönköping University, Jönköping, Sweden; 17 Division of Child & Adolescent Psychiatry, University of Cape Town, Cape Town, South Africa; 18 Department of Pediatrics, University of Alberta, Edmonton, Canada; 19 Child and Adolescent Psychiatry, Stockholm County Council, Stockholm, Sweden

**Keywords:** ASD, Neurodevelopmental disorder, Functioning, Assessment, ICD, DSM, Clinical study

## Abstract

This is the fourth international preparatory study designed to develop International Classification of Functioning, Disability and Health (ICF, and Children and Youth version, ICF-CY) Core Sets for Autism Spectrum Disorder (ASD). Examine functioning of individuals diagnosed with ASD as documented by the ICF-CY in a variety of clinical settings. A cross-sectional study was conducted, involving 11 units from 10 countries. Clinical investigators assessed functioning of 122 individuals with ASD using the ICF-CY checklist. In total, 139 ICF-CY categories were identified: 64 activities and participation, 40 body functions and 35 environmental factors. The study results reinforce the heterogeneity of ASD, as evidenced by the many functional and contextual domains impacting on ASD from a clinical perspective.

**Electronic supplementary material:**

The online version of this article (10.1007/s10803-018-3482-4) contains supplementary material, which is available to authorized users.

## Introduction

**Fig. 1:**
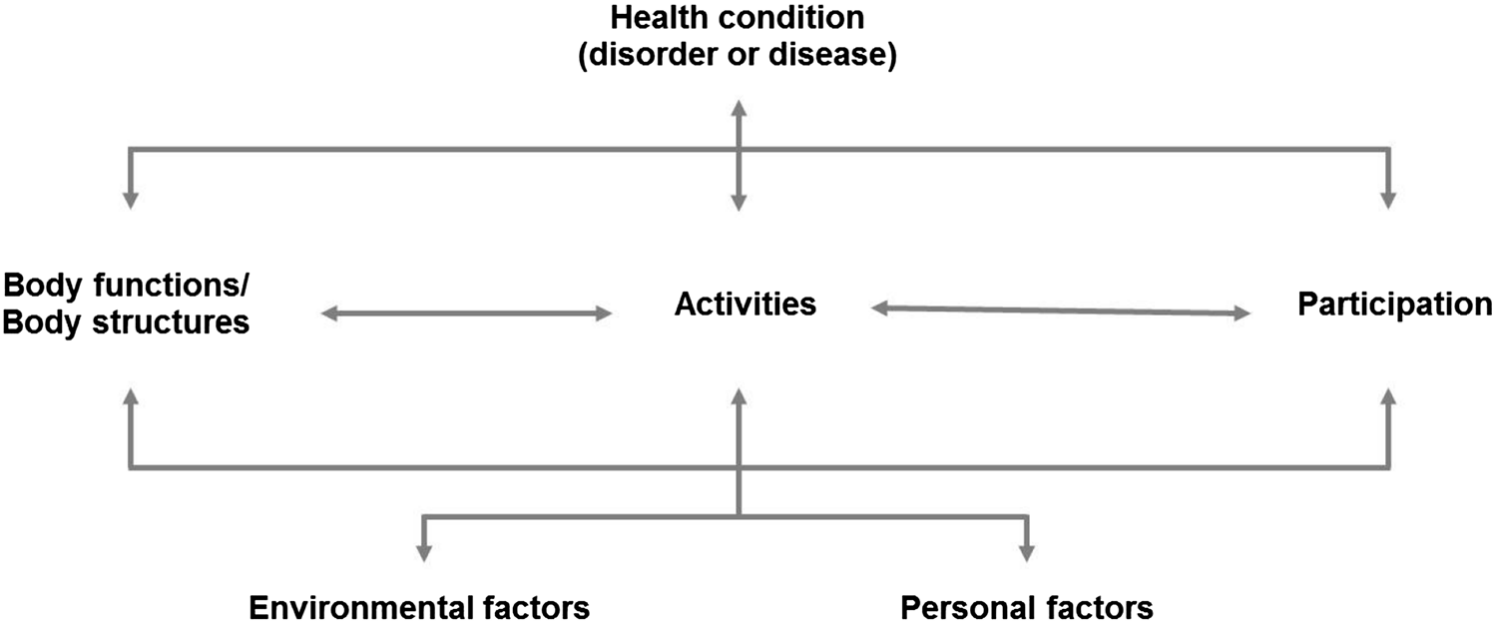
The ICF-CY is grounded on an interactive bio-psycho-social model of functioning. Independent of diagnosis, the ICF-CY provides detailed classifications in the components of body functions (i.e., physiological functions of body systems), body structures (i.e., anatomical parts of the body), activities (i.e., execution of tasks), participation (i.e., involvement in life situations), and environmental factors (i.e., physical, social and attitudinal environment). ICF-CY framework also includes personal factors that are inherent to the individual but not part of the individual’s primary health condition, such as gender, race/ethnicity, educational level and coping strategies. However, personal factors are not classified in the ICF-CY due to their large social and cultural variability (WHO 2001, 2007)

**Fig. 2:**
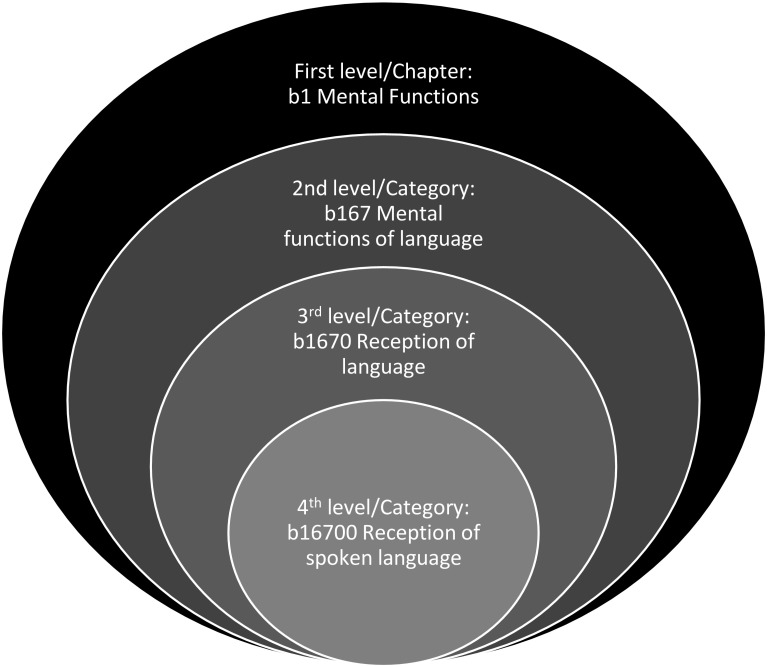
Each ICF-CY component is described and structured in four different levels of depth. The first level, referred to as “chapters”, provide a general overview of the areas of functioning and environment that are covered by the nomenclature. The chapters, in turn, consist of more specific categories of functioning and environment that are hierarchically structured with up to three levels of increasing detail, as demonstrated by the following body functions component example

Autism spectrum disorder (ASD) is a neurodevelopmental condition with an estimated worldwide prevalence of 1–2% (Baxter et al. 2015; CDC 2016; Idring et al. 2015) characterized by persistent difficulties in social communication and interaction, alongside restricted, repetitive behavior patterns and interests (APA 2013). The symptoms cause adverse functional outcomes in school (Levy and Perry 2011), work (Howlin et al. 2013), social relationships (Schmidt et al. 2015), domestic life (Fortuna et al. 2015; Matson et al. 2009) and self-care (Borremans et al. 2010; Du et al. 2015). ASD is also associated with an increased risk for other neurodevelopmental and psychiatric conditions (Pan 2014; Simonoff et al. 2008), alterations in physical health (Cashin et al. 2016; McElhanon et al. 2014), premature mortality (Hirvikoski et al. 2016) and lower life satisfaction (Jonsson et al. 2017; van Heijst and Geurts 2015). Even though ASD is primarily defined by challenges in various aspects of daily life, it has also been reported to entail specific strengths, such as attention to detail (Baron-Cohen et al. 2009; de Schipper et al. 2016), enhanced visuo-spatial skills (Happé and Frith 2009), creativity (de Schipper et al. 2016) and memory (de Schipper et al. 2016). Furthermore, environmental factors such as higher socio-economic background, parental commitment, and provision of evidence-based treatments, have found to facilitate the functional outcome of individuals with ASD regarding social relationships as well as school and vocational achievement (Delobel-Ayoub et al. 2015; Durkin et al. 2010; Kirby et al. 2016; Rai et al. 2012). The research findings here suggest that individual adaptive profile and composition of abilities and disabilities in ASD may vary substantially depending on developmental level, personal characteristics, access to service and other factors. Therefore, internationally accepted, standardized classification tools for individual assessment of functioning in individuals with ASD are desirable in clinical, research and educational settings. The International Classification of Functioning, Disability and Health (ICF) may serve as an effective framework for developing such tools. Officially endorsed by the World Health Organization in 2001, the ICF provides a comprehensive, internationally accepted nomenclature to describe health-related functioning in different conditions and condition groups, promoting an etiological-neutral perspective on disability (WHO 2001). In 2007, a child and youth version of the ICF (i.e., ICF-CY) was developed, specifically designed to capture functional abilities and disabilities in developing individuals by adding and expanding on the descriptions of existing ICF categories (WHO 2007). The ICF-CY is grounded on an interactive bio-psycho-social model of functioning (Fig. 1), which operationalizes functioning beyond medical or biological conception, taking into account other critical influences, such as the extended environment and a multitude of contextual factors (WHO 2001, 2007). Each component of ICF-CY comprises hierarchically structured categories that systematize various aspects of health-related functioning (Fig. 2).

The ICF-CY includes all ICF categories, plus additional ones specific to children and youth, making up 1685 categories in total (531 body functions, 329 body structures, 552 activities and participation categories, and 273 environmental factors) (WHO 2007). By providing a standard language for describing health and health-related states, the ICF-CY enables users to record useful profiles of individuals’ functioning across the lifespan for various purposes, ranging from diagnostic and treatment purposes (Bölte 2009; Escorpizo et al. 2013) to policy-making and raising public awareness of conditions (WHO 2007). However, using all categories from the ICF-CY to describe a specific health condition is unlikely to be meaningful, given that it will be time-consuming and often essentially undoable in clinical practice, as many categories would be irrelevant to specific conditions. To address this issue, the development of ICF Core Sets was initiated with the aim to allow user-friendly and effective descriptions of health-related functioning by generating shortlists of categories that are most relevant to specific health conditions. The development of ICF-CY “Core Sets” follows a rigorous scientific process, established and monitored by the WHO and the ICF Research Branch (Selb et al. 2015a), involving a wide range of professionals and stakeholders across all of the WHO-regions. The first phase of the project comprises four preparatory studies, each aiming to capture general and unique features of functioning and contextual factors specific to a certain health condition. The preparatory studies include a literature review (“research perspective”), an expert survey (“expert perspective”), a qualitative study (“client and close social environment perspective”), and a clinical study (“clinical perspective”). The present clinical study is therefore part of a superordinate project that will result in the development of standardized ICF Core Sets for ASD. As part of this project, ICF Core Sets are also being developed for attention deficit-hyperactivity disorder (ADHD), with the protocol and results reported in separate publications (Bölte et al. 2014; de Schipper et al. 2015). Once the preparatory studies have been completed, the results will be presented at an international consensus conference. At this conference, a group of ASD experts will review the findings from the preparatory studies and decide on which ICF-CY categories to include in the first official versions of the ICF Core Sets for the condition.

The objective of this study was to capture aspects of functioning and contextual factors pertaining to individuals with ASD as assessed by the ICF-CY in a clinical practice setting. For this purpose, an international cross-sectional multicenter study was conducted, involving clinicians and clinical researchers evaluating the functional level of children, adolescents and adults with ASD, as well as rating environmental barriers and facilitators.

## Method

### Procedure

The study was approved by the Regional Ethics Review Board in Stockholm and by the Local Ethics Review Boards at each of the other participating sites. Prior to study participation, informed written consent or assent was obtained from the diagnosed individuals and/or caregivers depending on age and cognitive and communication level. The consent form assured voluntarily study participation and confidentiality of the participants. An international cross-sectional, multi-center study design, as recommended by the WHO and ICF Research Branch (Selb et al. 2015a), was applied for this purpose, involving 11 clinical units from 10 countries across 4 WHO regions: Argentina (The Americas), Brazil (The Americas), Denmark (Europe), Germany (Europe), Greece (Europe), Italy (Europe), Japan (Western Pacific), Portugal (Europe), Saudi Arabia (Eastern Mediterranean) and Sweden (Europe). This composition of country representation was chosen for two primary reasons: (i) to meet the basic requirements of an international sample, and (ii) to enable future in-depth comparisons of cross-cultural perceptions of ASD, as these have shown to influence assessment and treatment of ASD (Burkett et al. 2015; Ratto et al. 2015). Participating sites were specialized in the clinical management of neurodevelopmental disorders.

Ratings were made based on information from medical records, medical history taking, neuropsychological testing and standardized clinical instrument scores (e.g., Wechsler Intelligence Scale for Children and Adults, Autism Diagnostic Observation Schedule, Child Behavior Checklist, Autism Diagnostic Interview-Revised, etc.), clinical observations and interviews with the participant and/or caregivers depending on age and developmental level of the rated case. The minimum dataset that was required for an individual to be included in this study was data from medical records and clinical observation. Clinicians and clinical researchers examined the medical information available at the respective site for each participant and extracted relevant information on socio-demography, co-morbidity and ASD-related functioning aspects. Co-morbidity was systematically assessed at all study sites by checking medical records. The investigators then proceeded to interview the participant and/or caregivers to rate the remaining categories of the ICF-CY checklist. Interviews varied in length from 40 to 120 min. Telephone interviews were occasionally used as an option to accommodate logistical challenges, but also to comply with some participants’ wishes to be interviewed via the phone due to ASD-related difficulties in face-to-face social interaction and communication.

### Participants

In total, N = 126 participants fulfilled criteria for participation and consented to take part in the study between March and August 2016. Inclusion criteria were a primary clinical diagnosis of ASD or a specified ASD diagnosis (autism, Asperger syndrome, atypical autism, pervasive developmental disorder not otherwise specified) along with any given common co-morbidity (if applicable) according to local or national guidelines and the diagnostic criteria of the ICD-10, DSM-IV/-TR or DSM-5 and/or receiving treatment for ASD. Participants were excluded from the study if the primary diagnosis was unclear or if the caregiver or the individual diagnosed with ASD could not communicate in the language of the country where the recruitment took place. Recruitment of participants was made at the respective clinical unit facilitated by the clinical investigators in charge. Nearly half of the adults (n = 18) and some preschool aged children (n = 8) were, however, recruited via local and national interest organizations for ASD. For most of these cases, access to medical records was limited and the rating of functioning level was primarily based on interview information. Following the steps of previous ICF clinical studies (Finger et al. 2011; Schiariti and Mâsse 2015), this study aimed to enroll at least 100 participants.

### Material

The ICF Checklist 2.1a version is a rating tool aimed to elicit and record information on the functioning and environment of an individual by using selected categories from the ICF (WHO 2003). The checklist consists of 123 second-level categories from the 4 ICF components (31 body functions, 12 body structures, 48 activities and participation, 32 environmental factors). In addition, the checklist also includes diagnostic information, which enables users to study the relationship between a health condition and associated functioning problems. The categories in the checklist are usually rated by using ICF qualifiers, a five-point scale that defines severity of functional impairment by looking at how often a specific problem is present in an individual’s daily life. The more often a specific problem is experienced, the larger the impact. The validity of the ICF checklist has been explored in previous studies (Ewert et al. 2004; Kohler et al. 2011; Okochi et al. 2005). The feasibility of the ICF checklist has also been shown in patients diagnosed with different kinds of chronic conditions (e.g., diabetes mellitus, osteoarthritis, ischemic heart disease, depressive disorder, etc.).

For the current study, a tailored version of the WHO ICF Checklist version 2.1a was used to rate the functional level of individuals with ASD (see Supplementary Material) and environmental barriers and facilitators. The checklist was divided into four parts. Part 1 contained the inclusion criteria of the study, part 2 included questions related to the socio-demographic background of the diagnosed individual, part 3 consisted of ratings of 161 second-level ICF-CY categories, and part 4 aimed to explore personal factors. To increase the specificity of the checklist content to individuals with ASD, 38 second-level ICF-CY categories were added in the checklist (17 body functions, 17 activities and participation categories, 3 environmental factors, and 1 body structure) based on results from our previous 3 preparatory studies: the literature review (Schipper et al. 2015, the expert survey Schipper et al. 2016 and the qualitative study Mahdi et al. 2017b). The 161 ICF-CY categories were distributed among all 4 ICF-CY components in the checklist and contained 65 activities and participation categories, 48 body functions, 35 environmental factors and 13 body structures.

An adapted version of the numeric rating scale (NRS) was used to rate each ICF-CY category in the checklist. The NRS (McCaffery and Beebe 1989), validated and commonly used to assess pain intensity (Ferreira-Valente et al. 2011), utilizes an 11-point scale, with 0 representing “no”, 1–3 “mild”, 4–6 “moderate” and 7–10 “severe” symptoms/impairment (McCaffery and Beebe 1989). In the current study, functional impairment and strengths were rated according to the NRS, following the same metrics as stated above. The main reason for using the NRS in this study was because of its relative simplicity and ease of administration and scoring (Ferreira-Valente et al. 2011). Contrary to ICF qualifiers, which define severity of functional impact by looking at how frequently a specific problem is experienced in daily life, the NRS does not offer a restricted definition. Instead, it enables investigators to explore other factors that may affect an individual’s functional level, such as degree and duration of impairment. ICF qualifiers have also been reported to be difficult to interpret by specific stakeholders, such as parents (Dalen et al. 2013). The NRS was also used to rate the categories in the environmental factors component, but with 0 representing “no barrier or facilitator”, + 10 “complete facilitator” and − 10 “complete barrier”. For all the components in the checklist, additional scoring options of “Not applicable” and “Not specified” were added. “Not applicable” was used if a specific ICF-CY category was not applicable to the individual (e.g., asking children about university or college studies), while the “Not specified” option was used if there was not sufficient information to rate the specific category. An option to capture strengths in ASD was also included. Strengths were defined as specific abilities that individuals with ASD were better at, compared to the average population. Information that indicated strengths in clinical observations, medical records or psychological test results were used to rate strengths. To minimize the possibility of over or underestimation of strengths (or difficulties) in interviews, the investigators were instructed to ask participants for clarifications and examples. Specific functioning categories that were not included in the checklist, but deemed important to ASD, were also documented and rated according to the NRS scale. An empty page was added in the checklist for the investigators to document any personal factors that were considered (either by the diagnosed individual or caregiver) to impact daily life functioning of individuals with ASD. Personal factors (e.g., gender, race, education level, specific life habits, etc.) were not rated, but documented descriptively in interviews with the participant and/or caregiver.

### Data Analysis

Any ICF-CY category that was rated as 2 or more in at least 10% of the cases was included as candidate category for the core set development. Although a scoring of “1” would be enough to classify a specific aspect of functioning or environmental factor as “mildly impaired/barrier/facilitator”, a more conservative cut-off was chosen to avoid margins of error (e.g., a specific problem might exist in daily life, but not be significantly impairing enough to affect functioning level). The choice of a 10% cut-off was based on results from previous ICF clinical studies (e.g., Vierhoff et al. 2015). The same cut-off was also applied to ratings that indicated above-average skills (strengths). Absolute (n) and relative (%) frequencies of difficulties and strengths were reported. Scorings that indicated “Not applicable” or “Not specified” were excluded from the frequency analyses. The participants’ socio-demographic background was summarized using descriptive statistics. Personal factors were analyzed exploratory by summarizing recurring themes.

### Quality Assurance

Prior to study participation, the lead investigator at each participating study site was required to take part in a web-based ICF self-learning course (http://icf.ideaday.de/). The course included an introduction to the ICF, its rationale and application areas. The aim of the course was to help the investigators understand the ICF model and classification terms that are used in the nomenclature. Another aim was to get the investigators to learn how to apply and use the ICF in practice. After completing the course, the investigators received examples of questions that they could use for the interviews with the participants. To get acquainted with the content of the ICF-CY checklist, each second-level ICF-CY category was provided with clear definitions and examples. Skype-meetings were arranged to discuss specific ICF-CY categories that were unclear. The checklist was translated into the language of each participating country, with the exception of Denmark, which used an English translation of the checklist. The study coordinator had regular contact with the study sites, monitoring the progress and providing material for quality management and comparability, such as sending interview experiences from other study sites, discussing ratings of ICF-CY categories.

### Sample

Of the 126 individuals who were eligible for participation, 122 completed the study. Attrition was due to not showing up for assessment (n = 3), or decline of participation without provision of a reason after initial written consent (n = 1). Table 1 shows the number of participants by country. Table 2 summarizes the socio-demographics of the participants included in the final analysis with respect to age, gender, marital status, education background, working status and living situation. Forty-five participants (37%) were diagnosed with ASD using DSM-5 criteria. Among the individuals who were diagnosed according to DSM-IV(-TR) or ICD-10 criteria, Asperger syndrome was diagnosed in 40 (33%) cases, followed by classic autism/autistic disorder (n = 26, 21%) and atypical autism/pervasive developmental disorder not otherwise specified (n = 11, 9%). The majority of the participants (n = 94, 77%) had at least one additional diagnosis. The most frequently reported co-morbidities were ADHD (n = 28, 23%), intellectual disability (n = 19, 16%), depression (n = 10, 8%), specific developmental disorder of motor function (n = 8, 7%) and generalized anxiety disorder (n = 5, 4%).

**Table 1:** Participants by county and WHO regions

Countries	WHO-regions	N (%)
Sweden	Europe	33 (27)
Germany (Dresden + Marburg)	Europe	26 (21)
Brazil	The Americas	15 (12)
Denmark	Europe	12 (10)
Saudi Arabia	Eastern Mediterranean	11 (9)
Greece	Europe	6 (5)
Italy	Europe	6 (5)
Japan	Western Pacific	6 (5)
Portugal	Europe	6 (5)
Argentina	The Americas	1 (1)

**Table 2:** Socio-demographic variables of diagnosed individuals

Socio-demographic variables	N (%)	Gender (female/male) N (%)	Age M (SD) Range
Age group			
Children with ASD (age 4–12)	46 (38)	10/36 (22/78)	8.3 (2.4) 4–12
Adolescents with ASD (age 13–17)	39 (32)	6/33 (15/85)	15.1 (1.5) 13–17
Adults with ASD (age 18+)	37 (30)	16/21 (43/57)	33.1 (10.7) 18–55
Marital status			
Single	112 (92)		
Divorced/separated	4 (4)		
Married	3 (2)		
Other^a^	3 (2)		
Education level^b^			
Primary/high school studies	81 (66)		
Vocational education	13 (11)		
Higher education (e.g., college or university studies)	10 (8)		
Other^c^	17 (14)		
Missing data	1 (1)		
Working status			
Student	80 (65)		
Supported employment	6 (5)		
Part-time employment	2 (2)		
Sickness-benefits	2 (2)		
Unemployment	2 (2)		
Combined forms of employment	15 (12)		
Other	15 (12)		
Living situation			
Living with parents	98 (81)		
Living independently	16 (13)		
Living with partner	3 (2)		
Other living situation^d^	4 (3)		
Combined living situations	1 (1)		

^a^Other marital status includes dating, live-apart, etc.

^b^One missing data for education level

^c^Other education level includes daycare, pre-school and folk high school

^d^Other living situation includes residential care living, living with a friend, etc.

## Results

### ICF-CY Category Ratings

In total, 139 of 161 ICF-CY categories assessed met the cut-off in at least 10% of the participants. Data saturation (Bowen 2008) showed that no candidate category would have been lost if data only were analyzed from Europe. There were, however, some candidate categories that would not have been covered in the non-European study sites. For example, 3 candidate categories (2%) were missing in the study sample that came from the Americas, while 15 (11%) were missing in Eastern Mediterranean and 48 (34%) in Western Pacific. The 139 candidate ICF-CY categories were distributed across 3 ICF-CY components: 64 categories in the activities and participation component, 40 body functions and 35 environmental factors. No body structure categories reached the cut-off. Table 3 shows the second-level categories identified in the activities and participation component, along with their absolute and relative frequencies. The categories were spread across all of the nine chapters in this component (Table 3), i.e., d1 learning and applying knowledge (k = 14), d4 mobility (k = 8), d5 self-care (k = 8), d7 interpersonal interactions and relationships (k = 7), d8 major life areas (k = 7), d3 communication (k = 6), d2 general demands and tasks (k = 5), d9 community, social and civic life (k = 5) and d6 domestic life (k = 4). The three most frequently identified ICF-CY categories in this component were d720 complex interpersonal interactions (n = 106, 86%), d710 basic interpersonal interactions (n = 104, 85%) and d240 handling stress and other psychological demands (n = 101, 82%).

**Table 3:** Absolute and relative frequencies of ICF-CY categories from the activities and participation component

Second-level category	ICF-CY chapter	n^a^ (%)
d110 watching	d1 Learning and applying knowledge	41 (33)
d115 listening	d1 Learning and applying knowledge	55 (45)
d130 copying	d1 Learning and applying knowledge	56 (45)
d132 acquiring information	d1 Learning and applying knowledge	55 (45)
d140 learning to read	d1 Learning and applying knowledge	36 (29)
d145 learning to write	d1 Learning and applying knowledge	37 (30)
d150 learning to calculate	d1 Learning and applying knowledge	44 (36)
d160 focusing attention	d1 Learning and applying knowledge	99 (81)
d161 directing attention	d1 Learning and applying knowledge	89 (72)
d163 thinking	d1 Learning and applying knowledge	58 (47)
d166 reading	d1 Learning and applying knowledge	48 (39)
d172 calculating	d1 Learning and applying knowledge	54 (44)
d175 solving problems	d1 Learning and applying knowledge	77 (63)
d177 making decisions	d1 Learning and applying knowledge	81 (66)
d210 undertaking a single task	d2 General tasks and demands	80 (65)
d220 undertaking multiple tasks	d2 General tasks and demands	86 (70)
d230 carrying out daily routine	d2 General tasks and demands	75 (61)
d240 handling stress and other psychological demands	d2 General tasks and demands	101 (82)
d250 managing one’s own behavior	d2 General tasks and demands	88 (72)
d310 communicating with receiving-spoken messages	d3 Communication	80 (65)
d315 communicating with receiving-nonverbal messages	d3 Communication	87 (71)
d330 speaking	d3 Communication	66 (54)
d335 producing nonverbal messages	d3 Communication	89 (72)
d350 conversation	d3 Communication	95 (77)
d360 using telecommunication devices and techniques	d3 Communication	51 (41)
d430 lifting and carrying objects	d4 Mobility	22 (18)
d440 fine hand use	d4 Mobility	51 (41)
d446 fine foot use	d4 Mobility	28 (22)
d450 walking	d4 Mobility	13 (10)
d455 moving around	d4 Mobility	27 (22)
d465 moving around using equipment	d4 Mobility	35 (28)
d470 using transportation	d4 Mobility	37 (30)
d475 driving	d4 Mobility	17 (13)
d510 washing oneself	d5 Self-care	50 (40)
d520 caring for body parts	d5 Self-care	66 (54)
d530 toileting	d5 Self-care	35 (28)
d540 dressing	d5 Self-care	43 (35)
d550 eating	d5 Self-care	30 (24)
d560 drinking	d5 Self-care	23 (18)
d570 looking after one’s health	d5 Self-care	71 (58)
d571 looking after one’s safety	d5 Self-care	54 (44)
d620 acquisition of goods and services	d6 Domestic life	47 (38)
d630 preparing meals	d6 Domestic life	61 (50)
d640 doing housework	d6 Domestic life	72 (59)
d660 assisting others	d6 Domestic life	61 (50)
d710 basic interpersonal interactions	d7 Interpersonal interactions and relationships	104 (85)
d720 complex interpersonal interactions	d7 Interpersonal interactions and relationships	106 (86)
d730 relating with strangers	d7 Interpersonal interactions and relationships	77 (63)
d740 formal relationships	d7 Interpersonal interactions and relationships	79 (64)
d750 informal social relationships	d7 Interpersonal interactions and relationships	92 (75)
d760 family relationships	d7 Interpersonal interactions and relationships	69 (56)
d770 intimate relationships	d7 Interpersonal interactions and relationships	30 (24)
d810 informal education	d8 Major life areas	39 (31)
d820 school education	d8 Major life areas	58 (47)
d845 acquiring, keeping and terminating a job	d8 Major life areas	23 (18)
d850 remunerative employment	d8 Major life areas	23 (18)
d860 basic economic transactions	d8 Major life areas	31 (25)
d870 economic self-sufficiency	d8 Major life areas	19 (15)
d880 engagement in play	d8 Major life areas	69 (56)
d910 community life	d9 Community, social and civic life	64 (52)
d920 recreation and leisure	d9 Community, social and civic life	83 (68)
d930 religion and spirituality	d9 Community, social and civic life	26 (21)
d940 human rights	d9 Community, social and civic life	32 (26)
d950 political life and citizenship	d9 Community, social and civic life	26 (21)

^a^Number of cases where the specific ICF-CY category was rated to be significantly affected by ASD

Frequencies of the second-level categories identified in the body functions component are listed in Table 4. The categories were identified in seven of the eight chapters in this component, i.e., b1 mental functions (k = 18), b2 sensory functions and pain (k = 8), b7 neuromusculoskeletal and movement-related functions (k = 5), b3 voice and speech functions (k = 3), b5 functions of the digestive, metabolic and endocrine systems (k = 3), b6 genitourinary and reproductive functions (k = 2) and b4 functions of the cardiovascular, haematological, immunological and respiratory systems (k = 1). The three most identified ICF-CY categories were b122 global psychosocial functions (n = 108, 88%), b125 dispositions and intra-personal functions (n = 106, 86%) and b140 attention functions (n = 105, 86%).

**Table 4:** Absolute and relative frequencies of ICF-CY categories from the body functions component

Second-level category	ICF-CY chapter	n^a^ (%)
b114 orientation functions	b1 Mental functions	50 (40)
b117 intellectual functions	b1 Mental functions	39 (31)
b122 global psychosocial functions	b1 Mental functions	108 (88)
b125 dispositions and intra-personal functions	b1 Mental functions	106 (86)
b126 temperament and personality functions	b1 Mental functions	95 (77)
b130 energy and drive functions	b1 Mental functions	89 (72)
b134 sleep functions	b1 Mental functions	48 (39)
b140 attention functions	b1 Mental functions	105 (86)
b144 memory functions	b1 Mental functions	48 (39)
b147 psychomotor functions	b1 Mental functions	70 (57)
b152 emotional functions	b1 Mental functions	100 (81)
b156 perceptual functions	b1 Mental functions	37 (30)
b160 thought functions	b1 Mental functions	56 (45)
b163 basic cognitive functions	b1 Mental functions	41 (33)
b164 higher-level cognitive functions	b1 Mental functions	91 (74)
b167 mental functions of language	b1 Mental functions	67 (54)
b172 calculation functions	b1 Mental functions	56 (45)
b180 experience of self and time functions	b1 Mental functions	70 (57)
b210 seeing functions	b2 Sensory functions and pain	21 (17)
b230 hearing functions	b2 Sensory functions and pain	32 (26)
b235 vestibular functions	b2 Sensory functions and pain	33 (27)
b250 taste functions	b2 Sensory functions and pain	17 (13)
b255 smell functions	b2 Sensory functions and pain	22 (18)
b265 touch function	b2 Sensory functions and pain	36 (29)
b270 sensory functions related to temperature and other stimuli	b2 Sensory functions and pain	39 (31)
b280 sensation of pain	b2 Sensory functions and pain	54 (44)
b310 voice functions	b3 Voice and speech functions	22 (18)
b320 articulation functions	b3 Voice and speech functions	34 (27)
b330 fluency and rhythm of speech functions	b3 Voice and speech functions	65 (53)
b435 immunological system functions	b4 Functions of the cardiovascular, hematological, immunological and respiratory systems	18 (14)
b515 digestive functions	b5 Functions of the digestive, metabolic and endocrine systems	14 (11)
b525 defecation functions	b5 Functions of the digestive, metabolic and endocrine systems	22 (18)
b530 weight maintenance functions	b5 Functions of the digestive, metabolic and endocrine systems	21 (17)
b620 urination functions	b6 Genitourinary and reproductive functions	14 (11)
b640 sexual functions	b6 Genitourinary and reproductive functions	14 (11)
b710 mobility of joint functions	b7 Neuromusculoskeletal and movement-related functions	14 (11)
b735 muscle tone functions	b7 Neuromusculoskeletal and movement-related functions	32 (26)
b760 control of voluntary movement functions	b7 Neuromusculoskeletal and movement-related functions	34 (27)
b765 involuntary movement functions	b7 Neuromusculoskeletal and movement-related functions	41 (33)
b770 gait pattern functions	b7 Neuromusculoskeletal and movement-related functions	22 (18)

^a^Number of cases where the specific ICF-CY category was rated to be significantly affected by ASD

Table 5 shows the frequencies of second-level categories that were identified in the environmental factors component. The categories in this component were identified in all five chapters, i.e., e5 services, systems and policies (k = 9), e3 support and relationships (k = 8), e4 attitudes (k = 8), e1 products and technology (k = 7) and e2 natural environment and human-made changes to environment (k = 3). The three most frequently identified second-level categories were e310 immediate family (n = 103, 84%), e410 individual attitudes of immediate family members (n = 93, 76%) and e355 health professionals (n = 87, 71%).

**Table 5:** Absolute and relative frequencies of ICF-CY categories from the environmental factors component

Second-level category	ICF-CY chapter	n^a^ (%)
e110 products or substances for personal consumption	e1 Products and technology	38 (31)
e115 products and technology for personal use in daily living	e1 Products and technology	59 (48)
e120 products and technology for personal indoor and outdoor mobility and transportation	e1 Products and technology	22 (18)
e125 products and technology for communication	e1 Products and technology	54 (44)
e150 design, construction and building products and technology of buildings for public use	e1 Products and technology	15 (12)
e155 design, construction and building products and technology of buildings for private use	e1 Products and technology	13 (10)
e165 assets	e1 Products and technology	14 (11)
e225 climate	e2 Natural environment and human-made changes to environment	31 (25)
e240 light	e2 Natural environment and human-made changes to environment	27 (23)
e250 sound	e2 Natural environment and human-made changes to environment	69 (56)
e310 immediate family	e3 Support and relationships	103 (84)
e315 extended family	e3 Support and relationships	52 (42)
e320 friends	e3 Support and relationships	47 (38)
e325 acquaintances, peers, colleagues, neighbors and community members	e3 Support and relationships	45 (36)
e330 people in positions of authority	e3 Support and relationships	56 (45)
e340 personal care providers and personal assistants	e3 Support and relationships	46 (37)
e355 health professionals	e3 Support and relationships	87 (71)
e360 other professionals	e3 Support and relationships	57 (46)
e410 individual attitudes of immediate family members	e4 Attitudes	93 (76)
e420 individual attitudes of friends	e4 Attitudes	39 (31)
e425 individual attitudes of acquaintances, peers, colleagues, neighbors and community members	e4 Attitudes	40 (32)
e440 individual attitudes of personal care providers and personal assistants	e4 Attitudes	38 (31)
e450 individual attitudes of health professionals	e4 Attitudes	73 (59)
e455 individual attitudes of other professionals	e4 Attitudes	44 (36)
e460 societal attitudes	e4 Attitudes	50 (40)
e465 social norms, practices and ideologies	e4 Attitudes	43 (35)
e525 housing services, systems and policies	e5 Services, systems and policies	27 (23)
e535 communication services, systems and policies	e5 Services, systems and policies	47 (38)
e540 transportation services, systems and policies	e5 Services, systems and policies	34 (27)
e550 legal services, systems and policies	e5 Services, systems and policies	36 (29)
e570 social security services, systems and policies	e5 Services, systems and policies	56 (45)
e575 general social support services, systems and policies	e5 Services, systems and policies	53 (43)
e580 health services, systems and policies	e5 Services, systems and policies	70 (57)
e585 education and training services, systems and policies	e5 Services, systems and policies	44 (36)
e590 labor and employment services, systems and policies	e5 Services, systems and policies	29 (24)

^a^Number of cases where the specific ICF-CY category was rated to be significantly relevant to ASD-related functioning

### ASD-Related Strengths

When analyzing ASD-related strengths, 3 ICF-CY categories met the cut-off of 2 in at least 10% of the participants. These included b144 memory functions (n = 20, 16%), d161 directing attention (n = 14, 11%) and b140 attention functions (n = 13, 10%).

### Personal Factors

The 122 cases yielded a total of 148 personal factors that were considered to either have a supportive or hampering impact on daily life functioning. The study sample showed a broad variation of personal factors. For this reason, personal factors were analyzed exploratively in order to investigate the data for any specific recurring themes. Examples of supportive personal factors included high IQ, acceptance towards own diagnosis and specific interests (e.g., art, sports). Having high IQ was mentioned to facilitate individuals in generating coping strategies to manage challenging or stressful life situations, while acceptance towards own diagnosis enabled individuals to seek knowledge and resources to understand their condition better, and thus adapt to their environment. Specific interests (e.g., art, sports) were reported to facilitate coming into contact with other people and improve their social interaction skills. Past traumatic life events (e.g., getting bullied at school) were mentioned as a hampering personal factor, as it affected the individual’s self-esteem and self-worth. Having caregivers with psychiatric disorders was also reported to negatively impact individual functioning, as it increased level of stress in daily life. Increased level of stress was in turn mentioned to exacerbate ASD symptoms. Perfectionism was another hampering personal factor that was mentioned to make it difficult to engage and initiate tasks and activities.

## Discussion

This international cross-sectional clinical study aimed to investigate functioning and contextual factors of individuals diagnosed with ASD using the ICF-CY framework. To achieve an international sample, participants with ASD were recruited from 11 clinical units from 10 countries and 4 WHO-regions. Not surprisingly, large number and broad variation of activities and participation categories were captured in this study, covering all nine chapters, ranging from difficulties with communication and social interaction to limitations in mobility, work, self-care and participation in civic life (including political and citizenship life). Although a rich variety of mental functions were captured in this study, other aspects of the body were also identified to be impacted by ASD, such as motor coordination deficits, hypersensitivity issues, gastrointestinal problems and voice and speech disfluency. Genitourinary and immunological functions were also considered to be affected by ASD. Environmental factors varied from support and attitudes of key individuals in life to provision of services and products and technology in daily living. Physical aspects of the environment, such as sound, climate, and light were also covered in this study. Strengths were scarcely reported in this study, but some recurring themes included memory (i.e., visuo-spatial long term-memory) and attention (i.e., hyper-focusing on tasks). Broad variation of personal factors was mentioned to affect functional level in ASD, including supportive factors such as high IQ and acceptance towards own diagnosis, as well as hampering factors, such as past traumatic life events and having family member with psychiatric disorder.

### Ratings of ICF-CY Categories

This study yielded a large number and variety of categories across 3 components and 21 ICF-CY chapters. Although the study findings suggest impairments in different cognitive functions, other aspects of the body were also found to be altered in ASD, such as motor coordination deficits (Fournier et al. 2010), gastrointestinal problems (McElhanon et al. 2014), voice and speech disfluency (Scaler Scott et al. 2014), and hypersensitivity issues (Marco et al. 2011). The same is true for immunological and genitourinary problems (Byers and Nichols 2014; Lyall et al. 2015). The results here underpin the importance of conducting multidisciplinary assessments in ASD to enable better treatment plans and prognosis by capturing all aspects of the body, including physical functions. The functional characteristics of ASD are further demonstrated by the fact that categories were identified from all nine chapters in the activities and participation component, corroborating previous research findings on difficulties in communication skills, social interaction, self-care, domestic life, and conductance of general tasks and demands (Borremans et al. 2010; Fortuna et al. 2015; Matson et al. 2009; Schmidt et al. 2015). We also consistently identified several functional aspects of ASD that have not been covered extensively by previous research, particularly regarding community and civic life, including participation in political and citizenship activities. The limitations detected were related to negative societal attitudes about the capacity of individuals with ASD to raise public awareness and actively engage in self-advocacy. Participants with ASD reported that they were not given a fair chance to engage in the public discourse on ASD. The latter might indicate violations of rights of individuals with disabilities to enjoy active participation in political life as committed in the UNICEF Convention on the Rights of Persons with Disabilities (CRPD). The need to make political participation more accessible to individuals with disabilities has previously been emphasized (Priestley et al. 2016). As the understanding of neurodevelopmental disorders is shifting with research advances challenging traditional notions of ASD, the voices of diagnosed individuals are essential to the public discourse on ASD (Wright et al. 2014). Another finding that deepen the current understanding of ASD was mobility, more specifically the usage of public motorized transportation, such as bus, train and metro. Research has shown that individuals with ASD face difficulties when using public transportation due to the absence of transportation options, lack of familiarity with public transportation, and cost factors (Lubin and Feeley 2016). However, we found that coping with sensory stimuli from the environment (e.g., noises, quick movements, strong scents) and stress caused by crowding during rush hour are experienced as the major challenges of public transport mobility. In general, we confirmed and identified a broad array of environmental factors being decisive for functioning in ASD. These environmental factors generate information on how individual functioning might be improved without changing the individual, but by using enhancing environmental facilitators and reducing barriers. Surprisingly, however, the role of environmental factors for functioning has largely been ignored in the diagnostic process of ASD, as evidenced by the fact that golden standard scales that are used to diagnose individuals with ASD do not sufficiently take into account environmental factors (Castro et al. 2013). Even more remarkably, they are underutilized in individual-based special education programs that aim to promote inclusive school curricula for individuals with ASD (Castro et al. 2012). The bio-psycho-social model of the ICF-CY can address this issue by generating comprehensive tools which professionals can use to explore environmental factors in-depth and as such facilitate interventions that meet the demands of individuals with ASD (WHO 2007). The ICF-CY stresses the responsibility of stakeholders to take an active role in modifying the environment to fit the needs of individuals with disabilities. The emphasis on environmental influences can provide the basis for interventions that are more inclusive and less stigmatizing for diagnosed individuals and their caregivers. Some issues, such as those concerning mobility (transportation) may for instance be easily addressed by offering alternatives (e.g., taxi, other time slots to avoid rush hours) or compensation (e.g., earplugs, assistance) that will allow individuals to attend to their daily errands and hobbies. Environmental factors can either functionally be perceived as a barrier or facilitator by the individual. For example, peer attitudes (i.e., e425 individual attitudes of acquaintances, peers, colleagues, neighbors and community members) may either be inclusive and lead to deeper social bonds or lead to discriminatory practices that cause social exclusion. The present study found individual support and attitudes to be essential to health-related functioning in ASD, which is in line with previous studies that have shown individual support and attitudes to influence school inclusion (Symes and Humphrey 2010) and successful work employment (Parr and Hunter 2014). Besides individual support and attitudes, large number of products and technology were considered relevant to the daily living of individuals with ASD, which is consistent with previous research findings, where technological aids have been used to improve communication skills in individuals with ASD (Ganz et al. 2012). Additionally, different types of services were identified to impact functioning in ASD, including services offered at health care, education, work and social settings. The findings here reinforce previous research findings, which suggest services to be provided at different levels and settings in order to optimize ASD outcome in daily life (Fein et al. 2017; Fleury et al. 2014; van Schalkwyk and Volkmar 2017). To improve outcome, the ICF-CY can jointly be used with the International Statistical Classification of Diseases–Tenth Revision (ICD-10) (WHO 1992) by complementing information on diseases, symptoms or complaints with data on how environmental factors influence daily life participation and execution of tasks (WHO 2007). These can also guide ICF-CY assessments recommended for ASD in ICD-11 (http://apps.who.int/classifications/icd11).

### ASD-Related Strengths

To date, this is the first international clinical study that investigated ASD-related strengths using the ICF-CY framework. Strengths reported here included memory and attention, which interestingly, were also commonly identified to be strengths in our previous expert survey of ASD (Schipper et al. 2016). Seriously taking into account strengths in ASD can be beneficial to enhance the functional outcomes of individuals with ASD. For example, attention to detail and intense focus have previously been found to increase work output among individuals with ASD (Smith et al. 1995). Their focus combined with their willingness to engage in repetitive and monotonous tasks may benefit employers, as these types of tasks are often disliked by others. Thus, they may provide valuable assistance to companies and organizations, while at the same time maintaining a more permanent position and become well-integrated in workplaces. Notably, several studies have found supervisors to rate their employee with ASD highly on a range of important job skills, suggesting that individuals with ASD can be successful in competitive, entry-level employment (Hillier et al. 2007). Exploring strengths can also help to balance-out deficit and resource-oriented views of ASD by facilitating interventions that are less stigmatizing and more focused on reinforcing already existing individual strengths. The ICF-CY can facilitate these types of interventions and strength assessments by not only capturing functional disabilities or limitations, but also individual strengths and abilities (WHO 2007).

### Study Limitations

This study faced some methodological issues that need to be considered in order to fully evaluate its validity. First, even though the assessed clinical sample involved cases from 10 different countries, the WHO-regions South East Asia (e.g., India) and Africa were, unfortunately, not covered. In addition, regarding the Western Pacific and The Americas, only the Far East and South America were covered, limiting potential inter-continental generalization. Second, a large portion of the study sample came from Europe, limiting cross-cultural comparisons and drawing of definite conclusions about ASD-related functioning from a global perspective. Although it is recommended by the WHO and ICF Research Branch (Selb et al. 2015a) to involve international stakeholders, it does not explicitly take into account exploration of cultural differences. Saturation analyses showed that all candidate categories were captured in study sites representing the WHO-region Europe. In other words, no candidate category would have been lost, if only data from Europe would have been analyzed. To ensure that the ICF-CY categories are universally representative, there is a need for cultural comparison studies that will explore this issue more extensively. Culture can play an important role in how quickly individuals with ASD get assessed and treated (Burkett et al. 2015; Ratto et al. 2015). For this reason, there are future plans on combining data from all preparatory investigations to explore cultural effects on ASD functioning more comprehensively with additional descriptive analyses. Indeed, conducting cross-cultural comparisons in the future may also add substantial value to the understanding of functioning from a global perspective (de Vries and Bölte 2016), since most knowledge and science of ASD originates from high-income countries, despite the fact that most people with ASD live in low to middle-income countries (Durkin et al. 2015). Nevertheless, the objective of this study was not to explore cultural influences on ASD-related functioning, but identify the most informative ICF-CY categories for ASD independent of culture from a clinical perspective. Another study limitation is that for some adult and preschool participants with ASD, there was no access to medical records, and thus ICF-CY assessment was primarily based on interview data. It is desirable in future studies to involve larger numbers of units being specialized in younger pediatric individuals and older adults diagnosed with ASD, as this study did not include large numbers of individuals with ASD in these age ranges. Furthermore, for some children and adolescents, interviews were only conducted with immediate family members. While proxy interviews are common in psychiatry in these age ranges, additional first-hand perspective interviews add substantial value. On the other hand, there are some challenges with including young individuals with disabilities. First, young individuals might lack the insight, communication skills or understanding to provide valid information (Jonsson et al. 2017). Second, children with developmental disabilities may experience difficulties with memory and recall, making it difficult for them to engage and fully respond to the interview questions (Coghill et al. 2009). Third, for children with mental health problems, disorder-specific symptoms and impairments may also hamper them from offering their own assessment (Danckaerts et al. 2010). For example, a child with ASD may experience difficulties with reporting on social relationships due to their limitations in verbal communication. Another study limitation is related to the lack of cases involving individuals with co-morbid intellectual disability, which potentially may have caused a biased representation of categories. However, given the large number and broad variation of candidate categories that were identified in this study, we expect the results to have covered the functional outcome of individuals from the entire autism spectrum. Further, gender and age group differences were not taken into account, partly because of the uneven representation of females and preschoolers with ASD, but also due to many confounder factors (e.g., culture, co-morbidity, ASD presentation, information sources) that might potentially cause biased results. A final limitation is that this study did not investigate inter-rater reliability between the investigators, mainly due to its international character with different languages used in the clinical work with patients at the respective sites. For compensation, the investigators were strictly encouraged to seek consensus ratings within their clinical teams pertaining to the cases.

## Conclusions

This clinical cross-sectional study sought to capture the entire spectrum of functioning in ASD from a clinical perspective, not only exploring disabilities, but also abilities and strengths, using the ICF-CY framework. In addition, environmental barriers and facilitators to individual functioning were comprehensively examined. The results from the current study are a step towards providing the scientific basis for developing ICF Core Sets for ASD, from which user-friendly tools can be derived and standardized for multi-purpose usage, ranging from preclinical and clinical research, educational and clinical practice to policy-making and service reimbursement models. These tools will facilitate in-depth assessments that enable diverse range of functional profiles to be captured, while at the same examine environmental facilitators and barriers to individual functioning across countries and WHO-regions. An integral part of the diagnostic process in the upcoming ICD-11 will be the usage of ICF-CY categories to assess the functional impact of a health condition (Selb et al. 2015b). The ICF Core Sets for ASD will provide stakeholders with shortlists of categories that are pertinent to ASD, thus complementing diagnostic information from golden standard scales and clinical observations with data on functional impact and environmental facilitators and barriers. The international framework of the ICF-CY also creates future possibilities to conduct cross-cultural comparisons, potentially adding substantial knowledge about daily life functioning and environment of those living with ASD in low to middle income countries. The data from the current study will along with results from the other preparatory studies provide the scientific basis for the decision-making process at the consensus conference, where the first version of the official ICF Core Sets for ASD will be determined.

## Electronic Supplementary material

Below is the link to the electronic supplementary material.

Supplementary material 1 (DOCX 198 KB)
