# Supplementary material for: An International Clinical Study of Ability and Disability in Autism Spectrum Disorder Using the WHO-ICF Framework
Source: J Autism Dev Disord. 2018 Feb 8;48(6):2148–63. doi: 10.1007/s10803-018-3482-4 (PMC5948258; doi:10.1007/s10803-018-3482-4)
Supplement: Supplementary file 1 — Supplementary material 1 (DOCX 198 KB) [file 10803_2018_3482_MOESM1_ESM.docx]

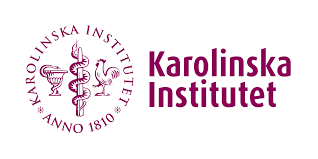

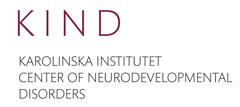


**Clinical cross-sectional study**

**Case record form for investigators**

**Use all available information when filling out the checklist. Mark with an “X” the information sources that have been used:**

[ ] Clinical observation [ ] Medical history

[ ] Interview with parent/caregiver [ ] Medical records

[ ] Interview with participant [ ] Tests/scales

**Name of investigator**

________________________________________________

**Working place of investigator**

**________________________________________________**

**Participant ID-number**

|  |
| --- |


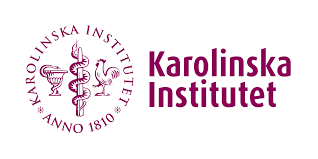

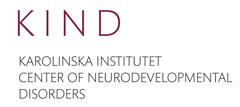


**Background about the study for investigators using this case record form**

Adaptive functioning and impairment are considered to be important components in clinical assessment and research study of health and quality of life in individuals with Autism Spectrum Disorder (ASD). In order to describe these aspects of functioning, the International Classification of Functioning, Disability and Health (ICF) was developed by the World Health Organization (WHO) with the aim to provide a comprehensive, universally accepted framework for the description of health-related functioning. There is also a children and youth version of the ICF, called ICF-CY (Children and Youth), that is specifically aimed at capturing functioning in developing individuals. The ICF-CY is divided into different components: body functions, body structures, activities (execution of task or action by individual), participation (involvement in a life situation) and environmental factors (factors in the environment in which people live and conduct their lives). Within each component, there are lists of categories that capture different aspects of functioning. Altogether, the ICF-CY includes more than 1600 categories of health-related functioning. Not all are, however, applicable to a certain health condition.

For this reason, the Karolinska Institutet, in cooperation with the ICF Research Branch, a cooperation partner within the WHO collaborating centre for the family of international classifications, has initiated the process of developing a specifically designed version of the ICF-CY (called ICF-CY Core Set) for individuals with ASD. The development of ICF-CY Core Set for ASD includes four different studies, each aiming to capture a specific perspective on functioning and health in ASD: a systematic literature review (research perspective), an expert survey (expert perspective), a focus group study (client/caregiver perspective) and a clinical cross-sectional study (clinician perspective), which is the current study.

This clinical study aims to investigate the perspective of the clinicians on ASD. Based on information from medical records (including medical history and results from tests/scales), clinical observations and interviews with participants/caregivers, clinicians will rate the functioning level of their clients using a checklist with categories from the ICF-CY. Information from the medical records can be used to rate most of the categories in the checklist. Results acquired from this study will be summarized and later published in a scientific journal.

Findings from this study, along with the ones from the other studies, will be presented in autumn 2016 at an international ICF consensus conference in Stockholm. At this conference a group of international ASD-experts will based on the results from the different studies determine which ICF-CY categories to be included in the Core Set for ASD. From the Core Set, different diagnostic tools (e.g. observation schedules, questionnaires, interviews) can be derived and standardized for broad use in clinical and research settings.

This study has been approved by the ethical review board in Stockholm.


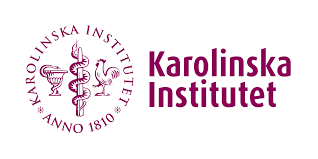

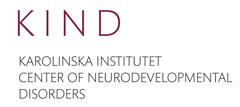


**Please follow these instructions when filling in the checklist:**

- Use a pen to fill in the checklist
- Answer all questions
- Write your answers as clearly as possible
- If you accidentally mark the wrong box, draw a single line through it and mark the correct box with an X. See the example below.

Example:

Yes  No

**
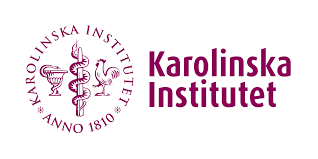

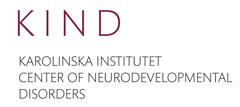
**

**Part 1: Inclusion criteria** (mark the correct box with an X)

**1. The participant has a primary DSM-IV or DSM-5 diagnosis of ASD Yes No**

- Asperger syndrome □
- Atypical autism □
- Classic autism/Autistic disorder □
- Autism Spectrum Disorder □

**2. The participant has been informed about the purpose and reason Yes No**

**of the study**

**3. The participant has signed the consent form Yes No**

**4. The parents of the participant have signed the consent form Yes No**

(if participants are unable to consent aged<16 years)

**
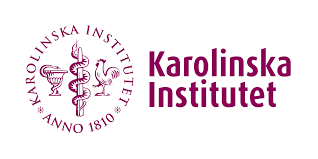

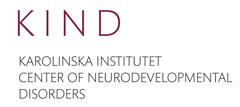
**

**Part 2: Information about the participant with ASD**

**1. Fill in the participant’s full date of birth (DD/MM/YYYY)**

|  |  | - |  |  | - |  |  |  |  |
| --- | --- | --- | --- | --- | --- | --- | --- | --- | --- |

**2. Mark the gender of the participant**

□ Female □ Male □ Other

**3. Mark the participant’s highest level of education**

□ Elementary school

□ High school

□ Higher education (e.g. college, university)

□ Vocational education

**4. Mark the participant’s marital status**

□ Single

□ Married

□ Living together with partner

□ Divorced/separated

□ Widower/widow

□ Other (please specify):________________________________________


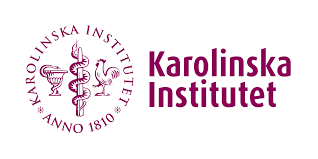

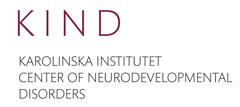


**5. Mark the participant’s living situation**

□ Living independently

□ Living with parents

□ Living with partner

□ Living in residential care

□ Other (please specify):___________________________________________

**6. Mark the participant’s current working status**

□ Full-time employment

□ Part-time employment

□ Volunteer work/unpaid employment

□ Supported employment

(Service provisions wherein people with disabilities, including mental health, are assisted with obtaining and maintaining employment through the primary models of job crews or coaches)

□ Unemployed

□ Student (elementary/high school, college, university, vocational education)

□ Receiving benefit grants (state benefits, e.g. disability grants, sickness grants, child care grants)

□ Other (please specify):______________________

**7. When did the participant receive his/her diagnosis (month, year)?**

|  |  | - |  |  |  |  |
| --- | --- | --- | --- | --- | --- | --- |

**8. Does the participant have other diagnoses in addition to ASD?** If yes, please specify.

__________________________________________________________________________________

__________________________________________________________________________________


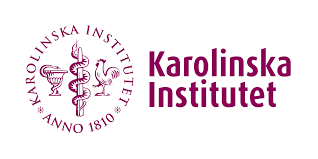

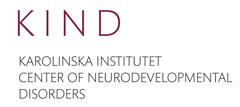


**Part 3: Introduction to the ICF-CY checklist for investigators**

This checklist consists of selected categories from the ICF-CY. The checklist is a practical tool that can be used to systematically gather information on functioning and impairment in individuals with ASD. The checklist is divided into different components, each aiming to describe functioning from a specific perspective. These are body functions, body structures, activities & participation and environmental factors. Within each component, there is an exhaustive list of categories that capture different aspects of functioning.

The checklist contains in total 161 ICF-CY categories; of which 123 were derived from WHO’s ICF Checklist; body functions = 48, body structures = 13, activities & participation = 65 and environmental factors = 35. To make the checklist more applicable to ASD, categories identified from previous ICF studies on ASD (systematic review, expert survey and focus group study) have been added to this checklist. Since the checklist consists of many functioning aspects, the investigators/users should be trained in the ICF-CY before they start rating the categories.

Instructions on how the categories are rated can be found for each component. Aspects of functioning that are not covered in the checklist but are considered to be relevant in ASD can also be included and rated in the checklist. The checklist is completed with a page about personal factors, in which users/investigators can add personal factors they believe to hamper or support the functioning level of the participant. Examples of personal factors include life style, habits, social background, education, important events in life etc.


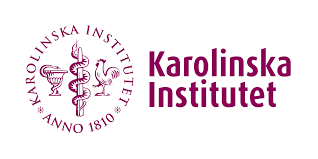

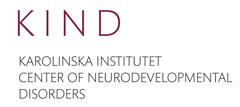


# Body functions component

In this part you will rate the level of impairment and strengths related to body function of the participant. For the rating, it is important to use all available information in the medical records (including results from tests/scales). Additional information is gathered through direct observation or interview with participant/caregiver. The information used for the rating can be up to three months old.

**Body functions** are the physiological functions of body systems (including physiological functions).

**Impairments** are problems in body function or structure as a significant deviation or loss.

**Strengths** are skills that are exhibited in a certain function of the body system (including physiological functions).

The categories in the body function component are rated on a scale from 0 to 10, in which **0 stands for “No impairment/no strength” and 10 for “Complete impairment/strength”.**

The code **“Not specified” (NS)** is used in cases where there is not enough information to specify the level of impairment/strength. In other words, there is impairment/strength but we don’t know the level of it.

The code **“Not applicable” (NA)** is used in cases where the specific code is not applicable to the participant, e.g. asking a question about family relationships when the participant does not have any family.

If a specific category is considered to be strength, first circle the option “Strength” and then do the scoring.


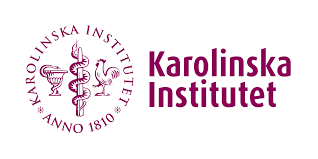

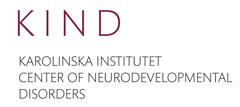


**0 = No impairment/strength 10 = Complete impairment/strength NS = Not specified NA = Not applicable**

| **b1 MENTAL FUNCTIONS** | **LEVEL OF FUNCTIONING** | | | | | | | | | | | |
| --- | --- | --- | --- | --- | --- | --- | --- | --- | --- | --- | --- | --- |
| **b110 Consciousness functions**  General mental functions of the state of awareness and alertness, including the clarity and continuity of the wakeful state. *Inclusions: functions of the state, continuity and quality of consciousness; loss of consciousness, coma, vegetative states, fugues, trance states, possession states, pharmacologically-(drug) induced altered consciousness, delirium, stupor.* | **0** | **1** | **2** | **3** | **4** | **5** | **6** | **7** | **8** | **9** | **10** | **NS NA** |
| **b114 Orientation functions**  General mental functions of knowing and ascertaining one’s relation to object, to self, to others, to time and to one’s surroundings and space. *Inclusions: functions of orientation in time, space, place and person; orientation to self and others; disorientation to time, place and person.* | **0** | **1** | **2** | **3** | **4** | **5** | **6** | **7** | **8** | **9** | **10** | **NS NA**  **Strength** |
| **b117 Intellectual functions**  General mental functions, required to understand and constructively integrate the various mental functions, including all cognitive functions and their development over the life span. *Inclusions: functions of intellectual growth; intellectual retardation, mental retardation, dementia.* | **0** | **1** | **2** | **3** | **4** | **5** | **6** | **7** | **8** | **9** | **10** | **NS NA**  **Strength** |
| **b122 Global psychosocial functions**  General mental functions, as they develop over the life span, required to understand and constructively integrate the mental functions that lead to the formation of the personal and interpersonal skills needed to establish reciprocal social interactions, in terms of both meaning and purpose. *Inclusion: any difficulty in self-other relationships including attachment.* | **0** | **1** | **2** | **3** | **4** | **5** | **6** | **7** | **8** | **9** | **10** | **NS NA** |
| **b125 Dispositions and intra-personal functions**  Disposition to act or react in a particular way, characterizing the personal, behavioural style of an individual that is distinct from others. These behavioural and responses styles are developmental in nature and may be foundational for later patterns of temperament and personality functions. *Inclusion: functions of adaptability, responsivity, activity level, predictability, persistence and approachability.* | **0** | **1** | **2** | **3** | **4** | **5** | **6** | **7** | **8** | **9** | **10** | **NS NA**  **Strength** |
| **b126 Temperament and personality functions**  General mental functions of constitutional disposition of the individual to react in a particular way to situations, including the set of mental characteristics that makes the individual distinct from others. *Inclusions: functions of extraversion, introversion, agreeableness, conscientiousness, psychic and emotional stability, and openness to experience; optimism; novelty seeking; confidence; trustworthiness.* | **0** | **1** | **2** | **3** | **4** | **5** | **6** | **7** | **8** | **9** | **10** | **NS NA**  **Strength** |


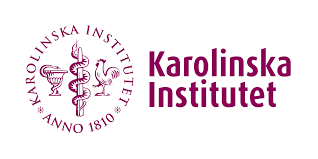

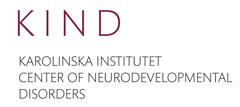


**0 = No impairment/strength 10 = Complete impairment/strength NS = Not specified NA = Not applicable**

| **b1 MENTAL FUNCTIONS** | **LEVEL OF FUNCTIONING** | | | | | | | | | | | |
| --- | --- | --- | --- | --- | --- | --- | --- | --- | --- | --- | --- | --- |
| **b130 Energy and drive functions**  General mental functions of physiological and psychological mechanisms that cause the individual to move towards satisfying specific needs and general goals in a persistent manner. *Inclusions: functions of energy level, motivation, appetite, craving (including craving for substances that can be abused), and impulse control.* | **0** | **1** | **2** | **3** | **4** | **5** | **6** | **7** | **8** | **9** | **10** | **NS NA**  **Strength** |
| **b134 Sleep functions**  General mental functions of periodic, reversible and selective physical and mental disengagement from one’s immediate environment accompanied by characteristic physiological changes. *Inclusions: functions of amount of sleeping, and onset, maintenance and quality of sleep; functions involving the sleep cycle, such as in insomnia, hypersomnia and narcolepsy.* | **0** | **1** | **2** | **3** | **4** | **5** | **6** | **7** | **8** | **9** | **10** | **NS NA** |
| **b140 Attention functions**  Specific mental functions of focusing on an external stimulus or internal experience for the required period of time. *Inclusions: functions of sustaining attention, shifting attention, dividing attention, sharing attention; concentration; distractibility.* | **0** | **1** | **2** | **3** | **4** | **5** | **6** | **7** | **8** | **9** | **10** | **NS NA**  **Strength** |
| **b144 Memory functions**  Specific mental functions of registering and storing information and retrieving it as needed. *Inclusions: functions of short-term, and long-term memory, immediate, recent and remote memory; memory span; retrieval of memory; remembering; functions used in recalling and learning, such as in nominal, selective and dissociative amnesia.* | **0** | **1** | **2** | **3** | **4** | **5** | **6** | **7** | **8** | **9** | **10** | **NS NA**  **Strength** |
| **b147 Psychomotor functions**  Specific mental functions of control over both motor and psychological events at the body level. *Inclusions: manual and lateral dominance functions of psychomotor control, such as in psychomotor delay, excitement and agitation, posturing, stereotypes, motor perseveration, catatonia, negativism, echopraxia and echolalia; quality of psychomotor function.* | **0** | **1** | **2** | **3** | **4** | **5** | **6** | **7** | **8** | **9** | **10** | **NS NA**  **Strength** |
| **b152 Emotional functions**  Specific mental functions related to the feeling and affective components of the processes of the mind. *Inclusions: functions of appropriateness of emotion, regulation and range of emotion; affect; sadness, happiness, love, fear, anger, hate, tension, anxiety, joy, sorrow; lability of emotion; flattening of affect.* | **0** | **1** | **2** | **3** | **4** | **5** | **6** | **7** | **8** | **9** | **10** | **NS NA**  **Strength** |
| **b156 Perceptual functions**  Specific mental functions of recognizing and interpreting sensory stimuli. *Inclusions: functions of auditory, visual, olfactory, gustatory, tactile and visuospatial perception, such as hallucination or illusion.* | **0** | **1** | **2** | **3** | **4** | **5** | **6** | **7** | **8** | **9** | **10** | **NS NA**  **Strength** |


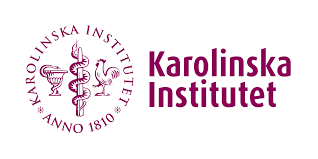

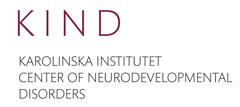


**0 = No impairment/strength 10 = Complete impairment/strength NS = Not specified NA = Not applicable**

| **b1 MENTAL FUNCTIONS** | **LEVEL OF FUNCTIONING** | | | | | | | | | | | |
| --- | --- | --- | --- | --- | --- | --- | --- | --- | --- | --- | --- | --- |
| **b160 Thought functions**  Specific mental functions related to the ideational component of the mind. *Inclusions: functions of pace, form, control and content of thought; goal-directed thought functions, non-goal directed thought functions; logical thought functions, such as pressure of thought, flight of ideas, thought block, incoherence of thought, circumstantiality, delusions, obsessions and compulsions.* | **0** | **1** | **2** | **3** | **4** | **5** | **6** | **7** | **8** | **9** | **10** | **NS NA**  **Strength** |
| **b163 Basic cognitive functions**  Mental functions involved in acquisition of knowledge about objects, events and experiences; and the organization and application of that knowledge in tasks requiring mental activity. *Inclusion: functions of cognitive development of representation, knowing and reasoning.* | **0** | **1** | **2** | **3** | **4** | **5** | **6** | **7** | **8** | **9** | **10** | **NS NA**  **Strength** |
| **b164 Higher-level cognitive functions**  Specific mental functions especially dependent on the frontal lobes of the brain, including complex goal-directed behaviours such as decision-making, abstract thinking, planning and carrying out plans, mental flexibility, and deciding which behaviours are appropriate under what circumstances; often called executive functions. *Inclusions: functions of abstraction and organization of ideas; time management, insight and judgement; concept formation, categorization and cognitive flexibility.* | **0** | **1** | **2** | **3** | **4** | **5** | **6** | **7** | **8** | **9** | **10** | **NS NA**  **Strength** |
| **b167 Mental functions of language**  Specific mental functions of recognizing and using signs, symbols and other components of a language. *Inclusions: functions of reception and decryption of spoken, written or other forms of language such as sign language; functions of expression of spoken, written or other forms of language; integrative language functions, spoken and written, such as involved in receptive, expressive, Broca’s, Wernicke’s and conduction aphasia.* | **0** | **1** | **2** | **3** | **4** | **5** | **6** | **7** | **8** | **9** | **10** | **NS NA**  **Strength** |
| **b172 Calculation functions**  Specific mental functions of determination, approximation and manipulation of mathematical symbols and processes. *Inclusions: functions of addition, subtraction, and other simple mathematical calculations; functions of complex mathematical operations.* | **0** | **1** | **2** | **3** | **4** | **5** | **6** | **7** | **8** | **9** | **10** | **NS NA**  **Strength** |
| **b180 Experience of self and time functions**  Specific mental functions related to the awareness of one’s identity, one’s body, one’s position in the reality of one’s environment and of time. *Inclusions: functions of experience of self, body image and time.* | **0** | **1** | **2** | **3** | **4** | **5** | **6** | **7** | **8** | **9** | **10** | **NS NA** |


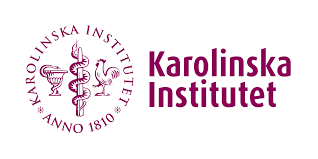

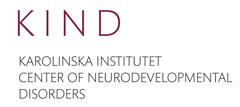


**0 = No impairment/strength 10 = Complete impairment/strength NS = Not specified NA = Not applicable**

| **b2 SENSORY FUNCTIONS AND PAIN** | **LEVEL OF FUNCTIONING** | | | | | | | | | | | |
| --- | --- | --- | --- | --- | --- | --- | --- | --- | --- | --- | --- | --- |
| **b210 Seeing functions**  Sensory functions relating to sensing the presence of light and sensing the form, size, shape and colour of the visual stimuli. *Inclusions: visual acuity functions; visual field functions; quality of vision; functions of sensing light and colour, visual acuity of distant and near vision, monocular and binocular vision; visual picture quality; impairments such as myopia, hypermetropia, astigmatism, hemianopia, colour-blindness, tunnel vision, central and peripheral scotoma, diplopia, night blindness and impaired adaptability to light.* | **0** | **1** | **2** | **3** | **4** | **5** | **6** | **7** | **8** | **9** | **10** | **NS NA**  **Strength** |
| **b230 Hearing functions**  Sensory functions relating to sensing the presence of sounds and discriminating the location, pitch, loudness and quality of sounds. *Inclusions: functions of hearing, auditory discrimination, localization of sound source, lateralization of sound, speech discrimination; impairments such as deafness, hearing impairment and hearing loss.* | **0** | **1** | **2** | **3** | **4** | **5** | **6** | **7** | **8** | **9** | **10** | **NS NA**  **Strength** |
| **b235 Vestibular functions**  Sensory functions of the inner ear related to position, balance and movement. *Inclusions: functions of position and positional sense; functions of balance of the body and movement.* | **0** | **1** | **2** | **3** | **4** | **5** | **6** | **7** | **8** | **9** | **10** | **NS NA**  **Strength** |
| **b250 Taste function**  Sensory functions of sensing qualities of bitterness, sweetness, sourness and saltiness. *Inclusions: gustatory functions, impairments such as ageusia and hypogeusia.* | **0** | **1** | **2** | **3** | **4** | **5** | **6** | **7** | **8** | **9** | **10** | **NS NA**  **Strength** |
| **b255 Smell function**  Sensory functions of sensing odours and smells. *Inclusions: olfactory functions; impairments such as anosmia or hyposmia.* | **0** | **1** | **2** | **3** | **4** | **5** | **6** | **7** | **8** | **9** | **10** | **NS NA**  **Strength** |
| **b265 Touch function**  Sensory functions of sensing surfaces and their texture or quality. *Inclusions: functions of touching, feeling of touch; impairments such as numbness, anaesthesia, tingling, paraesthesia and hyperaesthesia.* | **0** | **1** | **2** | **3** | **4** | **5** | **6** | **7** | **8** | **9** | **10** | **NS NA** |
| **b270 Sensory functions related to temperature and other stimuli**  Sensory functions of sensing temperature, vibration, pressure and noxious stimulus. *Inclusions: functions of being sensitive to temperature, vibration, shaking or oscillation, superficial pressure, deep pressure, burning sensation or a noxious stimulus.* | **0** | **1** | **2** | **3** | **4** | **5** | **6** | **7** | **8** | **9** | **10** | **NS NA** |
| **b280 Sensation of pain**  Sensation of unpleasant feeling indicating potential or actual damage to some body structure. *Inclusions: sensations of generalized or localized pain, in one or more body part, pain in a dermatome, stabbing pain, burning pain, dull pain, aching pain; impairments such as myalgia, analgesia and hyperalgesia.* | **0** | **1** | **2** | **3** | **4** | **5** | **6** | **7** | **8** | **9** | **10** | **NS NA** |


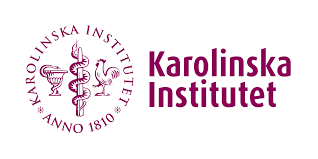

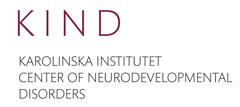


**0 = No impairment/strength 10 = Complete impairment/strength NS = Not specified NA = Not applicable**

| **b3 VOICE AND SPEECH FUNCTIONS** | **LEVEL OF FUNCTIONING** | | | | | | | | | | | |
| --- | --- | --- | --- | --- | --- | --- | --- | --- | --- | --- | --- | --- |
| **b310 Voice functions**  Functions of the production of various sounds by the passage of air through the larynx. *Inclusions: functions of production and quality of voice; functions of phonation, pitch, loudness and other qualities of voice; impairments such as aphonia, dysphonia, hoarseness, hypernasality and hyponasality.* | **0** | **1** | **2** | **3** | **4** | **5** | **6** | **7** | **8** | **9** | **10** | **NS NA** |
| **b320 Articulation functions**  Functions of the production of speech sounds. *Inclusions: functions of enunciation, articulation of phonemes; spastic, ataxic, flaccid dysarthria, anarthria.* | **0** | **1** | **2** | **3** | **4** | **5** | **6** | **7** | **8** | **9** | **10** | **NS NA** |
| **b330 Fluency and rhythm of speech functions**  Functions of the production of flow and tempo of speech. *Inclusions: functions of fluency, rhythm, speed and melody of speech; prosody and intonation; impairments such as stuttering, stammering, cluttering, bradylalia and tachylalia.* | **0** | **1** | **2** | **3** | **4** | **5** | **6** | **7** | **8** | **9** | **10** | **NS NA** |
| **b4 FUNCTIONS OF THE CARDIOVASCULAR,**  **HAEMATOLOGICAL, IMMUNOLOGICAL AND**  **RESPIRATORY SYSTEMS** | **LEVEL OF FUNCTIONING** | | | | | | | | | | | |
| **b410 Heart functions**  Functions of pumping the blood in adequate or required amounts and pressure throughout the body. *Inclusions: functions of heart rate, rhythm and output; contraction force of ventricular muscles; functions of heart valves; pumping the blood through the pulmonary circuit; dynamics of circulation to the heart; impairments such as tachycardia, bradycardia and irregular heart beat and as in heart failure, cardiomyopathy, myocarditis, and coronary insufficiency.* | **0** | **1** | **2** | **3** | **4** | **5** | **6** | **7** | **8** | **9** | **10** | **NS NA** |
| **b420 Blood pressure functions**  Functions of maintaining the pressure of blood within the arteries. *Inclusions: functions of maintenance of blood pressure; increased and decreased blood pressure; impairments such as in hypotension, hypertension and postural hypotension.* | **0** | **1** | **2** | **3** | **4** | **5** | **6** | **7** | **8** | **9** | **10** | **NS NA** |
| **b430 Haematological system functions**  Functions of blood production, oxygen and metabolite carriage, and clotting. *Inclusions: functions of the production of blood and bone marrow; oxygen-carrying functions of blood; blood-related functions of spleen; metabolite-carrying functions of blood; clotting; impairments such as anaemia, haemophilia and other clotting dysfunctions.* | **0** | **1** | **2** | **3** | **4** | **5** | **6** | **7** | **8** | **9** | **10** | **NS NA** |


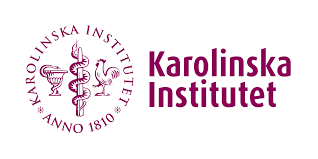

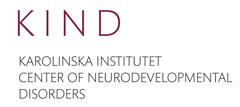


**0 = No impairment/strength 10 = Complete impairment/strength NS = Not specified NA = Not applicable**

| **b4 FUNCTIONS OF THE CARDIOVASCULAR,**  **HAEMATOLOGICAL, IMMUNOLOGICAL AND**  **RESPIRATORY SYSTEMS** | **LEVEL OF FUNCTIONING** | | | | | | | | | | | |
| --- | --- | --- | --- | --- | --- | --- | --- | --- | --- | --- | --- | --- |
| **b435 Immunological system functions**  Functions of the body related to protection against foreign substances, including infections, by specific and non-specific immune responses. *Inclusions: immune response (specific and non-specific); hypersensitivity reactions; functions of lymphatic vessels and nodes; functions of cell-mediated immunity, antibody-mediated immunity; response to immunization; impairments such as in autoimmunity,allergic reactions, lymphadenitis and lymphoedema.* | **0** | **1** | **2** | **3** | **4** | **5** | **6** | **7** | **8** | **9** | **10** | **NS NA** |
| **b440 Respiration functions**  Functions of inhaling air into the lungs, the exchange of gases between air and blood, and exhaling air. *Inclusions: functions of respiration rate, rhythm and depth; impairments such as apnoea, hyperventilation, irregular respiration, paradoxical respiration, and brochial spasm, and as in pulmonary emphysema; upper pulmonary obstruction, reduction in airflow through upper and lower airways.* | **0** | **1** | **2** | **3** | **4** | **5** | **6** | **7** | **8** | **9** | **10** | **NS NA** |
| **b5 FUNCTIONS OF THE DIGESTIVE, METABOLIC**  **AND ENDOCRINE SYSTEMS** | **LEVEL OF FUNCTIONING** | | | | | | | | | | | |
| **b510 Ingestion functions**  Functions related to taking in and manipulating solids or liquids through the mouth into the body. *Inclusions: functions of sucking, chewing and biting, manipulating food in the mouth, salivation, swallowing, burping, regurgitation, spitting and vomiting; impairments such as dysphagia, aspiration of food, aerophagia, excessive salivation, drooling and insufficient salivation.* | **0** | **1** | **2** | **3** | **4** | **5** | **6** | **7** | **8** | **9** | **10** | **NS NA** |
| **b515 Digestive functions**  Functions of transporting food through the gastrointestinal tract, breakdown of food and absorption of nutrients. *Inclusions: functions of transport of food through the stomach, peristalsis; breakdown of food, enzyme production and action in stomach and intestines; absorption of nutrients and tolerance to food; impairments such as in hyperacidity of stomach, malabsorption, intolerance to food, hypermotility of intestines, intestinal paralysis, intestinal obstruction and decreased bile production.* | **0** | **1** | **2** | **3** | **4** | **5** | **6** | **7** | **8** | **9** | **10** | **NS NA** |
| **b525 Defecation functions**  Functions of elimination of wastes and undigested food as faeces and related functions. *Inclusions: functions of elimination, faecal consistency, frequency of defecation; faecal continence, flatulence; impairments such as constipation, diarrhea, watery stool and anal sphincter incompetence or incontinence.* | **0** | **1** | **2** | **3** | **4** | **5** | **6** | **7** | **8** | **9** | **10** | **NS NA** |


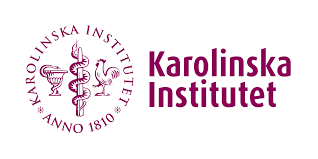

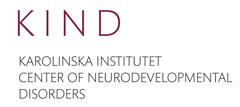


**0 = No impairment/strength 10 = Complete impairment/strength NS = Not specified NA = Not applicable**

| **b5 FUNCTIONS OF THE DIGESTIVE, METABOLIC**  **AND ENDOCRINE SYSTEMS** | **LEVEL OF FUNCTIONING** | | | | | | | | | | | |
| --- | --- | --- | --- | --- | --- | --- | --- | --- | --- | --- | --- | --- |
| **b530 Weight maintenance functions**  Functions of maintaining appropriate body weight, including weight gain during the developmental period. *Inclusions: functions of maintenance of acceptable Body Mass Index (BMI); and impairments such as underweight, cachexia, wasting, overweight, emaciation and such as in primary and secondary obesity.* | **0** | **1** | **2** | **3** | **4** | **5** | **6** | **7** | **8** | **9** | **10** | **NS NA** |
| **b555 Endocrine gland functions**  Functions of production and regulation of hormonal levels in the body, including cyclical changes. *Inclusions: functions of hormonal balance; hyperpituitarism, hypopituitarism, hyperthyroidism, hypothyroidism, hyperadrenalism, hypoadrenalism, hyperparathyroidism, hypoparathyroidism, hypergonadism, hypogonadism.* | **0** | **1** | **2** | **3** | **4** | **5** | **6** | **7** | **8** | **9** | **10** | **NS NA** |
| **b6 GENITOURINARY AND REPRODUCTIVE**  **FUNCTIONS** | **LEVEL OF FUNCTIONING** | | | | | | | | | | | |
| **b620 Urination functions**  Functions of discharge of urine from the urinary bladder. *Inclusions: functions of urination, frequency of urination, urinary continence; impairments such as in stress, urge, reflex, overflow, continuous incontinence, dribbling, automatic bladder, polyuria, urinary retention and urinary urgency.* | **0** | **1** | **2** | **3** | **4** | **5** | **6** | **7** | **8** | **9** | **10** | **NS NA** |
| **b640 Sexual functions**  Mental and physical functions related to the sexual act, including the arousal, preparatory, orgasmic and resolution stages. *Inclusions: functions of the sexual arousal, preparatory, orgasmic and resolution phase: functions related to sexual interest, performance, penile erection, clitoral erection, vaginal lubrication, masturbation, ejaculation, orgasm; impairments such as impotence, frigidity, vaginismus, premature ejaculation, priapism and delayed ejaculation.* | **0** | **1** | **2** | **3** | **4** | **5** | **6** | **7** | **8** | **9** | **10** | **NS NA** |
| **b7 NEUROMUSCULOSKELETAL AND MOVEMENT-**  **RELATED FUNCTIONS** | **LEVEL OF FUNCTIONING** | | | | | | | | | | | |
| **b710 Mobility of joint functions**  Functions of the range and ease of movement of a joint. *Inclusions: functions of mobility of single or several joints, vertebral, shoulder, elbow, wrist, hip, knee, ankle, small joints of hands and feet; mobility of joints generalized; impairments such as in hypermobility of joints, frozen joints, frozen shoulder, arthritis.* | **0** | **1** | **2** | **3** | **4** | **5** | **6** | **7** | **8** | **9** | **10** | **NS NA** |
|  |  |  |  |  |  |  |  |  |  |  |  |  |


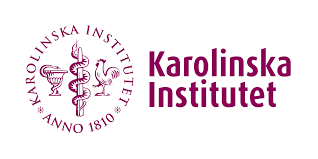

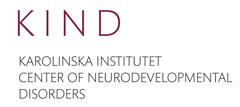


**0 = No impairment/strength 10 = Complete impairment/strength NS = Not specified NA = Not applicable**

| **b7 NEUROMUSCULOSKELETAL AND MOVEMENT-**  **RELATED FUNCTIONS** | **LEVEL OF FUNCTIONING** | | | | | | | | | | | |
| --- | --- | --- | --- | --- | --- | --- | --- | --- | --- | --- | --- | --- |
| **b730 Muscle power functions**  Functions related to the force generated by the contraction of a muscle or muscle groups. *Inclusions: functions associated with the power of specific muscles and muscle groups, muscles of one limb, one side of the body , the lower half of the body, all limbs, the trunk and the body as a whole; impairments such as weakness of small muscles in feet and hands, muscle paresis, muscle paralysis, monoplegia, hemiplegia, paraplegia, quadriplegia and akinetic mutism.* | **0** | **1** | **2** | **3** | **4** | **5** | **6** | **7** | **8** | **9** | **10** | **NS NA**  **Strength** |
| **b735 Muscle tone functions**  Functions related to the tension present in the resting muscles and the resistance offered when trying to move the muscles passively. *Inclusions: functions associated with the tension of isolated muscles and muscle groups, muscles of one limb, one side of the body and the lower half of the body, muscles of all limbs, muscles of the trunk, and all muscles of the body; impairments such as hypotonia, hypertonia and muscle spasticity, myotonia and paramyotonia.* | **0** | **1** | **2** | **3** | **4** | **5** | **6** | **7** | **8** | **9** | **10** | **NS NA** |
| **b760 Control of voluntary movement functions**  Functions associated with control over and coordination of voluntary movements. *Inclusions: functions of control of simple voluntary movements and of complex voluntary movements, coordination of voluntary movements, supportive functions of arm or leg, right left motor coordination, eye hand coordination, eye foot coordination; impairments such as control and coordination problems, e.g. clumsiness and dysdiadochokinesia.* | **0** | **1** | **2** | **3** | **4** | **5** | **6** | **7** | **8** | **9** | **10** | **NS NA**  **Strength** |
| **b765 Involuntary movement functions**  Functions of unintentional, non-or semi-purposive involuntary contractions of a muscle or group of muscles. *Inclusions: involuntary contractions of muscles; impairments such as tremors, tics, mannerisms, stereotypies, motor perseveration, chorea, athetosis, vocal tics, dystonic movements and dyskinesia.* | **0** | **1** | **2** | **3** | **4** | **5** | **6** | **7** | **8** | **9** | **10** | **NS NA** |
| **b770 Gait pattern functions**  Functions of movement patterns associated with walking, running or other whole body movements. *Inclusions: walking patterns and running patterns; impairments such as spastic gait, hemiplegic gait, paraplegic gait, asymmetric gait, limping and stiff gait pattern.* | **0** | **1** | **2** | **3** | **4** | **5** | **6** | **7** | **8** | **9** | **10** | **NS NA** |


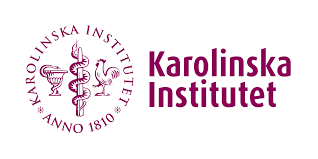

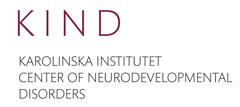


**0 = No impairment/strength 10 = Complete impairment/strength NS = Not specified NA = Not applicable**

| **ANY OTHER BODY FUNCTIONS** | **LEVEL OF FUNCTIONING** | | | | | | | | | | | |
| --- | --- | --- | --- | --- | --- | --- | --- | --- | --- | --- | --- | --- |
|  | **0** | **1** | **2** | **3** | **4** | **5** | **6** | **7** | **8** | **9** | **10** | **NS NA**  **Strength** |
|  | **0** | **1** | **2** | **3** | **4** | **5** | **6** | **7** | **8** | **9** | **10** | **NS NA**  **Strength** |
|  | **0** | **1** | **2** | **3** | **4** | **5** | **6** | **7** | **8** | **9** | **10** | **NS NA**  **Strength** |
|  | **0** | **1** | **2** | **3** | **4** | **5** | **6** | **7** | **8** | **9** | **10** | **NS NA**  **Strength** |
|  | **0** | **1** | **2** | **3** | **4** | **5** | **6** | **7** | **8** | **9** | **10** | **NS NA**  **Strength** |
|  | **0** | **1** | **2** | **3** | **4** | **5** | **6** | **7** | **8** | **9** | **10** | **NS NA**  **Strength** |
|  | **0** | **1** | **2** | **3** | **4** | **5** | **6** | **7** | **8** | **9** | **10** | **NS NA**  **Strength** |
|  | **0** | **1** | **2** | **3** | **4** | **5** | **6** | **7** | **8** | **9** | **10** | **NS NA**  **Strength** |
|  | **0** | **1** | **2** | **3** | **4** | **5** | **6** | **7** | **8** | **9** | **10** | **NS NA**  **Strength** |


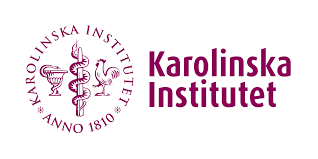

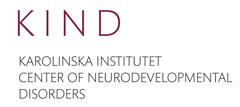


# Body structures component

In this part you will rate the level of impairment and strengths related to body structure of the participant. For the rating, it is important to use all available information in the medical records. Additional information is gathered through direct observation or interview with participant/caregiver. The information used for the rating can be up to three months old.

**Body structures** are anatomical parts of the body, such as organs, limbs and their components.

**Impairments** are problems in body structure, such as significant deviation or loss.

**Strengths** are significant deviations in body structure that enables the individual to function well.

The categories in the body structures component are rated on a scale from 0 to 10, in which **0 stands for “No impairment/strength” and 10 for “Complete impairment/strength”.**

The code **“Not specified” (NS)** is used in cases where there is not enough information to specify the level of impairment/strength. In other words, there is impairment/strength but we don’t know the level of it.

The code **“Not applicable” (NA)** is used in cases where the specific code is not applicable to the participant, e.g. asking a question about family relationships when the participant does not have any family.

If a specific category is considered to be strength, first circle the option “Strength” and then do the scoring.


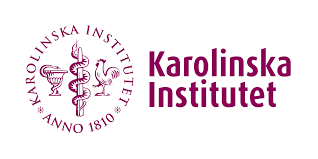

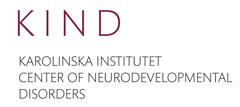


**0 = No impairment/strength 10 = Complete impairment/strength NS = Not specified NA = Not applicable**

| **s1 STRUCTURES OF THE NERVOUS SYSTEM** | **LEVEL OF FUNCTIONING** | | | | | | | | | | | |
| --- | --- | --- | --- | --- | --- | --- | --- | --- | --- | --- | --- | --- |
| **s110 Structure of brain**  *Inclusions: structure of cortical lobes, structure of cerebellum, structure of midbrain, structure of brain stem, structure of cranial nerves and structure of white matter.* | **0** | **1** | **2** | **3** | **4** | **5** | **6** | **7** | **8** | **9** | **10** | **NS NA**  **Strength** |
| **s120 Spinal cord and related structures** | **0** | **1** | **2** | **3** | **4** | **5** | **6** | **7** | **8** | **9** | **10** | **NS NA** |
| **s3 STRUCTURES INVOLVED IN VOICE AND SPEECH** | **LEVEL OF FUNCTIONING** | | | | | | | | | | | |
| **s320 Structure of mouth**  *Inclusions: teeth, gums, structure of palate, tongue, structure of lips, structure of pharynx and structure of larynx.* | **0** | **1** | **2** | **3** | **4** | **5** | **6** | **7** | **8** | **9** | **10** | **NS NA** |
| **s4 STRUCTURES OF THE CARDIOVASCULAR,**  **IMMUNOLOGICAL AND RESPIRATORY SYSTEMS** | **LEVEL OF FUNCTIONING** | | | | | | | | | | | |
| **s410 Structure of cardiovascular system**  *Inclusions: heart (atria, ventricles), arteries, veins, capillaries).* | **0** | **1** | **2** | **3** | **4** | **5** | **6** | **7** | **8** | **9** | **10** | **NS NA** |
| **s430 Structure of respiratory system**  *Inclusions: trachea, lungs, thoracic cage and muscles of respiration.* | **0** | **1** | **2** | **3** | **4** | **5** | **6** | **7** | **8** | **9** | **10** | **NS NA** |
| **s6 STRUCTURES RELATED TO THE GENITOURINARY AND REPRODUCTIVE SYSTEMS** | **LEVEL OF FUNCTIONING** | | | | | | | | | | | |
| **s610 Structure of urinary system**  *Inclusions: kidneys, ureters, urinary bladder, urethra.* | **0** | **1** | **2** | **3** | **4** | **5** | **6** | **7** | **8** | **9** | **10** | **NS NA** |
| **s630 Structure of reproductive system**  *Inclusions: ovaries, uterus, breast and nipple, structure of vagina and external genitalia, testes and scrotum, peni.* | **0** | **1** | **2** | **3** | **4** | **5** | **6** | **7** | **8** | **9** | **10** | **NS NA** |
| **s7 STRUCTURES RELATED TO MOVEMENT** | **LEVEL OF FUNCTIONING** | | | | | | | | | | | |
| **s710 Structure of head and neck region**  *Inclusions: bones of cranium, bones of face, bones of neck region, joints of head and neck region, muscles of head and neck region, ligaments and fasciae of head and neck region.* | **0** | **1** | **2** | **3** | **4** | **5** | **6** | **7** | **8** | **9** | **10** | **NS NA** |
| **s720 Structure of shoulder region**  *Inclusions: bones of shoulder region, joints of shoulder region, muscles of shoulder region, ligaments and fasciae of shoulder region.* | **0** | **1** | **2** | **3** | **4** | **5** | **6** | **7** | **8** | **9** | **10** | **NS NA** |
| **s730 Structure of upper extremity**  *Inclusions: structure of upper arm, structure of forearm, structure of hand.* | **0** | **1** | **2** | **3** | **4** | **5** | **6** | **7** | **8** | **9** | **10** | **NS NA**  **Strength** |
| **s740 Structure of pelvic region**  *Inclusions: bones of pelvic region, joints of pelvic region, muscles of pelvic region, ligaments and fasciae of pelvic region.* | **0** | **1** | **2** | **3** | **4** | **5** | **6** | **7** | **8** | **9** | **10** | **NS NA** |


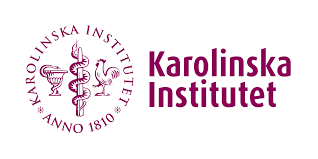

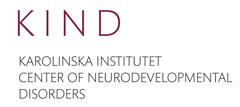


**0 = No impairment/strength 10 = Complete impairment/strength NS = Not specified NA = Not applicable**

| **s7 STRUCTURES RELATED TO MOVEMENT** |  | **LEVEL OF FUNCTIONING** | | | | | | | | | | |
| --- | --- | --- | --- | --- | --- | --- | --- | --- | --- | --- | --- | --- |
| **s750 Structure of lower extremity**  *Inclusions: structure of thigh, structure of lower leg, structure of ankle and foot.* | **0** | **1** | **2** | **3** | **4** | **5** | **6** | **7** | **8** | **9** | **10** | **NS NA**  **Strength** |
| **s760 Structure of trunk**  *Inclusions: structure of vertebral column, muscles of trunk, ligaments and fasciae of trunk.* | **0** | **1** | **2** | **3** | **4** | **5** | **6** | **7** | **8** | **9** | **10** | **NS NA** |


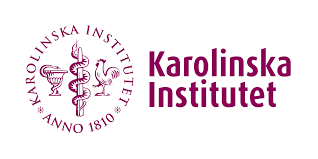

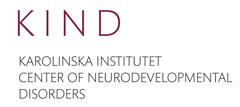


**Activities & Participation component**

In this part you will rate the level of limitation/restriction and skills related to everyday life activity and participation of the individual. For the rating, it is important to use all available information in the medical records. Additional information is gathered through direct observation or interview with participant/caregiver. The information used for the rating can be up to three months old.

**Activity** is the execution of a task or action by an individual.

**Activity limitations** are difficulties an individual may have in executing activities.

**Participation** is involvement in a life situation.

**Participation restrictions** are problems an individual may experience in involvement in life situations.

During childhood and adolescence, limitations and restrictions may also take the form of delays or lags in the emergence of activities and participation.

**Strengths** are skills that are exhibited in a certain activity (e.g. “doing housework”) or participation in major life area (e.g. “school”).

The categories in the activities and participation component are rated on a scale from 0 to 10, in which **0 stands for “No difficulty/strength” and 10 for “Complete difficulty/strength”.**

The code **“Not specified” (NS)** is used in cases where there is not enough information to specify the level of impairment/strength. In other words, there is impairment/strength but we don’t know the level of it.

The code **“Not applicable” (NA)** is used in cases where the specific code is not applicable to the participant, e.g. asking a question about family relationships when the participant does not have any family.

If a specific category is considered to be strength, first circle the option “Strength” and then do the scoring.


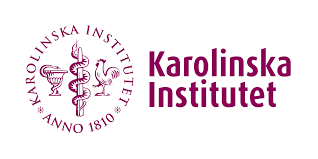

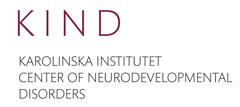


**0 = No difficulty/strength 10 = Complete difficulty/strength NS = Not specified NA = Not applicable**

| **d1 LEARNING AND APPLYING KNOWLEDGE** | **LEVEL OF FUNCTIONING** | | | | | | | | | | | |
| --- | --- | --- | --- | --- | --- | --- | --- | --- | --- | --- | --- | --- |
| **d110 Watching**  Using the sense of seeing intentionally to experience visual stimuli, such as visually tracking an object, watching persons, looking at a sporting event, person, or children playing. | **0** | **1** | **2** | **3** | **4** | **5** | **6** | **7** | **8** | **9** | **10** | **NS NA**  **Strength** |
| **d115 Listening**  Using the sense of hearing intentionally to experience auditory stimuli, such as listening to a radio, the human voice, to music, a lecture, or to a story told. | **0** | **1** | **2** | **3** | **4** | **5** | **6** | **7** | **8** | **9** | **10** | **NS NA**  **Strength** |
| **d130 Copying**  Imitating or mimicking as a basic component of learning, such as copying, repeating a facial expression, a gesture, a sound or the letters of an alphabet. *Inclusion: immediate imitation of an action or behavior.* | **0** | **1** | **2** | **3** | **4** | **5** | **6** | **7** | **8** | **9** | **10** | **NS NA**  **Strength** |
| **d132 Acquiring information**  Obtaining facts about persons, things and events, such as asking why, what, where and how, asking for names. | **0** | **1** | **2** | **3** | **4** | **5** | **6** | **7** | **8** | **9** | **10** | **NS NA**  **Strength** |
| **d140 Learning to read**  Developing the competence to read written material (including Braille and other symbols) with fluency and accuracy, such as recognizing characters and alphabets, sounding out written words with correct pronunciation, and understanding words and phrases. | **0** | **1** | **2** | **3** | **4** | **5** | **6** | **7** | **8** | **9** | **10** | **NS NA**  **Strength** |
| **d145 Learning to write**  Developing the competence to produce symbols that represent sounds, words or phrases in order to convey meaning (including Braille writing and other symbols), such as spelling effectively and using correct grammar. | **0** | **1** | **2** | **3** | **4** | **5** | **6** | **7** | **8** | **9** | **10** | **NS NA**  **Strength** |
| **d150 Learning to calculate**  Developing the competence to manipulate numbers and perform simple and complex mathematical operations, such as using mathematical signs for addition and subtraction and applying the correct mathematical operation to a problem. | **0** | **1** | **2** | **3** | **4** | **5** | **6** | **7** | **8** | **9** | **10** | **NS NA**  **Strength** |
| **d160 Focusing attention**  Intentionally focusing on specific stimuli, such as by filtering out distracting noises. | **0** | **1** | **2** | **3** | **4** | **5** | **6** | **7** | **8** | **9** | **10** | **NS NA**  **Strength** |
| **d161 Directing attention**  Intentionally maintaining attention to specific actions or tasks for an appropriate length of time. | **0** | **1** | **2** | **3** | **4** | **5** | **6** | **7** | **8** | **9** | **10** | **NS NA**  **Strength** |
| **d163 Thinking**  Formulating and manipulating ideas, concepts, and images, whether goal-oriented or not, either alone or with others, with types of thinking activities, such as pretending, playing with words, creating fiction, proving a theorem, playing with ideas, brainstorming, meditating, pondering, speculating or reflecting. | **0** | **1** | **2** | **3** | **4** | **5** | **6** | **7** | **8** | **9** | **10** | **NS NA**  **Strength** |


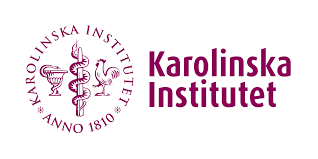

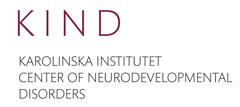


**0 = No difficulty/strength 10 = Complete difficulty/strength NS = Not specified NA = Not applicable**

| **d1 LEARNING AND APPLYING KNOWLEDGE** | **LEVEL OF FUNCTIONING** | | | | | | | | | | | |
| --- | --- | --- | --- | --- | --- | --- | --- | --- | --- | --- | --- | --- |
| **d166 Reading**  Performing activities involved in the comprehension and interpretation of written language (e.g. books, instructions, newspapers in text or Braille), for the purpose of obtaining general knowledge or specific information. *Inclusion: comprehension and interpretation of written language in standard form of letters or characters as well as text created with unique symbols such as icons.* | **0** | **1** | **2** | **3** | **4** | **5** | **6** | **7** | **8** | **9** | **10** | **NS NA**  **Strength** |
| **d172 Calculating**  Performing computations by applying mathematical principles to solve problems that are described in words and producing or displaying the results, such as computing the sum of three numbers or finding the result of dividing one number by another. | **0** | **1** | **2** | **3** | **4** | **5** | **6** | **7** | **8** | **9** | **10** | **NS NA**  **Strength** |
| **d175 Solving problems**  Finding solutions to questions or situations by identifying and analyzing issues, developing options and solutions evaluating potential effects of solutions, and executing a chosen solution such as in resolving a dispute between two people. | **0** | **1** | **2** | **3** | **4** | **5** | **6** | **7** | **8** | **9** | **10** | **NS NA**  **Strength** |
| **d177 Making decisions**  Making a choice among options, implementing the choice, and evaluating the effects of the choice, such as selecting and purchasing a specific item, or deciding to undertake and undertaking one task among several tasks that need to be done. | **0** | **1** | **2** | **3** | **4** | **5** | **6** | **7** | **8** | **9** | **10** | **NS NA**  **Strength** |
| **d2 GENERAL TASKS AND DEMANDS** | **LEVEL OF FUNCTIONING** | | | | | | | | | | | |
| **d210 Undertaking a single task**  Carrying out simple or complex and coordinated actions related to the mental and physical components of a single task, such as initiating a task, organizing time, space and materials for a task, pacing task performance, and carrying out, completing and sustaining a task. *Inclusions: undertaking a simple or complex task; undertaking a single task independently or in a group.* | **0** | **1** | **2** | **3** | **4** | **5** | **6** | **7** | **8** | **9** | **10** | **NS NA**  **Strength** |
| **d220 Undertaking multiple tasks**  Carrying out simple or complex and coordinated actions as components of multiple, integrated and complex tasks in sequence or simultaneously. *Inclusions: undertaking multiple tasks; completing multiple tasks; undertaking multiple tasks independently and in a group.* | **0** | **1** | **2** | **3** | **4** | **5** | **6** | **7** | **8** | **9** | **10** | **NS NA**  **Strength** |
| **d230 Carrying out daily routine**  Carrying out simple or complex and coordinated actions in order to plan, manage and complete the requirements of day-to-day procedures or duties, such as budgeting time and making plans for separate activities throughout the day. *Inclusions: managing and completing the daily routine; managing one’s own activity level.* | **0** | **1** | **2** | **3** | **4** | **5** | **6** | **7** | **8** | **9** | **10** | **NS NA**  **Strength** |


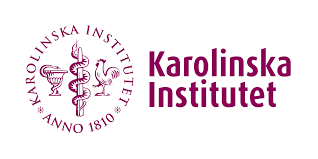

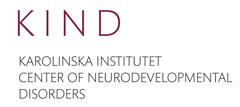


**0 = No difficulty/strength 10 = Complete difficulty/strength NS = Not specified NA = Not applicable**

| **d2 GENERAL TASKS AND DEMANDS** | **LEVEL OF FUNCTIONING** | | | | | | | | | | | |
| --- | --- | --- | --- | --- | --- | --- | --- | --- | --- | --- | --- | --- |
| **d240 Handling stress and other psychological demands**  Carrying out simple or complex and coordinated actions to manage and control the psychological demands required to carry out tasks demanding significant responsibilities and involving stress, distraction, or crises, such as taking exams, driving a vehicle during heavy traffic, putting on clothes when hurried by parents, finishing a task within a time-limit or taking care of a large group of children.*Inclusions: handling responsibilities; handling stress and crisis.* | **0** | **1** | **2** | **3** | **4** | **5** | **6** | **7** | **8** | **9** | **10** | **NS NA**  **Strength** |
| **d250 Managing one’s own behaviour**  Carrying out simple or complex and coordinated actions in a consistent manner in response to new situations, persons or experiences, such as being quiet in a library. | **0** | **1** | **2** | **3** | **4** | **5** | **6** | **7** | **8** | **9** | **10** | **NS NA**  **Strength** |
| **d3 COMMUNICATION** | **LEVEL OF FUNCTIONING** | | | | | | | | | | | |
| **d310 Communicating with –receiving –spoken messages**  Comprehending literal and implied meanings of messages in spoken language, such as understanding that a statement asserts a fact or is an idiomatic expression, such as responding and comprehending spoken messages. | **0** | **1** | **2** | **3** | **4** | **5** | **6** | **7** | **8** | **9** | **10** | **NS NA**  **Strength** |
| **d315 Communicating with –receiving –nonverbal messages**  Comprehending the literal and implied meanings of messages conveyed by gestures, symbols and drawings, such as realizing that a child is tired when she rubs her eyes or that a warning bell means that there is a fire. *Inclusions: communicating with –receiving –body gestures, general signs and symbols, drawings and photographs.* | **0** | **1** | **2** | **3** | **4** | **5** | **6** | **7** | **8** | **9** | **10** | **NS NA**  **Strength** |
| **d330 Speaking**  Producing words, phrases and longer passages in spoken messages with literal and implied meaning, such as expressing a fact or telling a story in oral language. | **0** | **1** | **2** | **3** | **4** | **5** | **6** | **7** | **8** | **9** | **10** | **NS NA**  **Strength** |
| **d335 Producing nonverbal messages**  Using gestures, symbols and drawings to convey messages, such as shaking one’s head to indicate disagreement or drawing a picture or diagram to convey a fact or complex idea. *Inclusions: producing body gestures, signs, symbols, drawings and photographs.* | **0** | **1** | **2** | **3** | **4** | **5** | **6** | **7** | **8** | **9** | **10** | **NS NA**  **Strength** |
| **d350 Conversation**  Starting, sustaining and ending an interchange of thoughts and ideas, carried out by means of spoken, written, sign or other forms of language, with one or more persons one knows or who are strangers, in formal and casual settings. *Inclusions: starting, sustaining and ending a conversation; conversing with one or many people.* | **0** | **1** | **2** | **3** | **4** | **5** | **6** | **7** | **8** | **9** | **10** | **NS NA**  **Strength** |


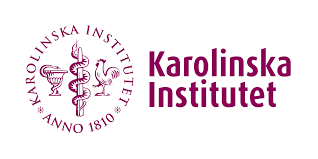

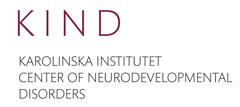


**0 = No difficulty/strength 10 = Complete difficulty/strength NS = Not specified NA = Not applicable**

| **d3 COMMUNICATION** | **LEVEL OF FUNCTIONING** | | | | | | | | | | | |
| --- | --- | --- | --- | --- | --- | --- | --- | --- | --- | --- | --- | --- |
| **d360 Using communication devices and techniques**  Using devices, techniques and other means for the purposes of communicating, such as calling a friend on the telephone. *Inclusions: using telecommunication devices, using writing machines and communication techniques.* | **0** | **1** | **2** | **3** | **4** | **5** | **6** | **7** | **8** | **9** | **10** | **NS NA**  **Strength** |
| **d4 MOBILITY** | **LEVEL OF FUNCTIONING** | | | | | | | | | | | |
| **d430 Lifting and carrying objects**  Raising up an object or taking something from one place to another, such as when lifting a cup or toy, or carrying a box or a child from one room to another. *Inclusions: lifting, carrying in the hands or arms, or on shoulders, hip, back or head; putting down.* | **0** | **1** | **2** | **3** | **4** | **5** | **6** | **7** | **8** | **9** | **10** | **NS NA**  **Strength** |
| **d440 Fine hand use**  Performing the coordinated actions of handling objects, picking up, manipulating and releasing them using one’s hand, fingers and thumb, such as required to lift coins off a table or turn a dial or knob. *Inclusions: picking up, grasping, manipulating and releasing.* | **0** | **1** | **2** | **3** | **4** | **5** | **6** | **7** | **8** | **9** | **10** | **NS NA**  **Strength** |
| **d446 Fine foot use**  Performing the coordinated actions to move or manipulate objects using one’s foot. | **0** | **1** | **2** | **3** | **4** | **5** | **6** | **7** | **8** | **9** | **10** | **NS NA**  **Strength** |
| **d450 Walking**  Moving along a surface on foot, step by step, so that one foot is always on the ground, such as when strolling, sauntering, walking forwards, backwards, or sideways. *Inclusions: walking short or long distances; walking on different surfaces; walking around obstacles.* | **0** | **1** | **2** | **3** | **4** | **5** | **6** | **7** | **8** | **9** | **10** | **NS NA**  **Strength** |
| **d455 Moving around**  Moving the whole body from one place to another by means other than walking, such as climbing over a rock or running down a street, skipping, scampering, jumping, somersaulting or running around obstacles. | **0** | **1** | **2** | **3** | **4** | **5** | **6** | **7** | **8** | **9** | **10** | **NS NA**  **Strength** |
| **d465 Moving around using equipment**  Moving the whole body from place to place, on any surface or space, by using specific devices designed to facilitate moving or create other ways of moving around, such as with skates, skis, scuba equipment, swim fins, or moving down the street in a wheelchair or a walker. | **0** | **1** | **2** | **3** | **4** | **5** | **6** | **7** | **8** | **9** | **10** | **NS NA**  **Strength** |
| **d470 Using transportation**  Using transportation to move around as a passenger, such as being driven in a car, bus, rickshaw, jitney, pram or stroller, animal-powered vehicle, private or public taxi, train, tram, subway, boat or aircraft. *Inclusions: using human-powered transportation; using private motorized or public transportation.* | **0** | **1** | **2** | **3** | **4** | **5** | **6** | **7** | **8** | **9** | **10** | **NS NA** |


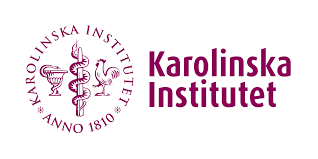

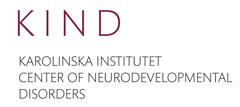


**0 = No difficulty/strength 10 = Complete difficulty/strength NS = Not specified NA = Not applicable**

| **d4 MOBILITY** | **LEVEL OF FUNCTIONING** | | | | | | | | | | | |
| --- | --- | --- | --- | --- | --- | --- | --- | --- | --- | --- | --- | --- |
| **d475 Driving**  Being in control of and moving a vehicle or the animal that draws it, travelling under one’s own direction or having at one’s disposal any form of transportation, such as a car, bicycle, boat or animal-powered vehicle. *Inclusions: driving human-powered transportation, motorized vehicles, animal-powered vehicles.* | **0** | **1** | **2** | **3** | **4** | **5** | **6** | **7** | **8** | **9** | **10** | **NS NA**  **Strength** |
| **d5 SELF CARE** | **LEVEL OF FUNCTIONING** | | | | | | | | | | | |
| **d510 Washing oneself**  Washing and drying one’s whole body, or body parts, using water and appropriate cleaning and drying materials or methods, such as bathing, showering, washing hands and feet, face and hair, and drying with a towel. *Inclusions: washing body parts, the whole body; and drying oneself.* | **0** | **1** | **2** | **3** | **4** | **5** | **6** | **7** | **8** | **9** | **10** | **NS NA**  **Strength** |
| **d520 Caring for body parts**  Looking after those parts of the body, such as skin, face, teeth, scalp, nails and genitals, that require more than washing and drying. *Inclusions: caring for skin, teeth, hair, finger and toe nails, and nose.* | **0** | **1** | **2** | **3** | **4** | **5** | **6** | **7** | **8** | **9** | **10** | **NS NA**  **Strength** |
| **d530 Toileting**  Indicating the need for, planning and carrying out the elimination of human waste (menstruation, urination and defecation), and cleaning oneself afterwards. *Inclusions: regulating urination, defecation and menstrual care* | **0** | **1** | **2** | **3** | **4** | **5** | **6** | **7** | **8** | **9** | **10** | **NS NA** |
| **d540 Dressing**  Carrying out the coordinated actions and tasks of putting on and taking off clothes and footwear in sequence and in keeping with climatic and social conditions, such as by putting on, adjusting and removing shirts, skirts, blouses, pants, undergarments, saris, kimono, tights, hats, gloves, coats, shoes, boots, sandals and slippers. *Inclusions: putting on or taking off clothes and footwear and choosing appropriate clothing.* | **0** | **1** | **2** | **3** | **4** | **5** | **6** | **7** | **8** | **9** | **10** | **NS NA** |
| **d550 Eating**  Indicating need for, and carrying out the coordinated tasks and actions of eating food that has been served, bringing it to the mouth and consuming it in culturally acceptable ways, cutting or breaking food into pieces, opening bottles and cans, using eating implements, having meals, feasting or dining. | **0** | **1** | **2** | **3** | **4** | **5** | **6** | **7** | **8** | **9** | **10** | **NS NA** |
| **d560 Drinking**  Indicating need for, and taking hold of a drink, bringing it to the mouth and consuming the drink in culturally acceptable ways; mixing, stirring and pouring liquids for drinking, opening bottles and cans, drinking through a straw or drinking running water, such as from a tap or a spring; feeding from the breast. | **0** | **1** | **2** | **3** | **4** | **5** | **6** | **7** | **8** | **9** | **10** | **NS NA** |


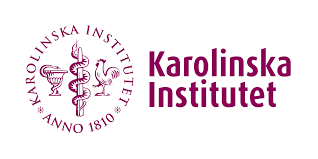

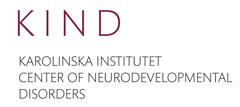


**0 = No difficulty/strength 10 = Complete difficulty/strength NS = Not specified NA = Not applicable**

| **d5 SELF CARE** | **LEVEL OF FUNCTIONING** | | | | | | | | | | | | |
| --- | --- | --- | --- | --- | --- | --- | --- | --- | --- | --- | --- | --- | --- |
| **d570 Looking after one’s health**  Ensuring or indicating needs about physical comfort, health and physical and mental well-being, such as by maintaining a balanced diet and an appropriate level of physical activity, keeping warm or cool, avoiding harm to health, following safe sex practices, including using condoms, getting immunizations and regular physical examinations. *Inclusions: ensuring one’s physical comfort; managing diet and fitness; maintaining one’s health.* | **0** | **1** | **2** | **3** | **4** | **5** | **6** | **7** | **8** | **9** | **10** | **NS NA**  **Strength** | |
| **d571 Looking after one’s safety**  Avoiding risks that can lead to physical injury or harm. Avoiding potentially hazardous situations such as misusing fire or running into traffic. | **0** | **1** | **2** | **3** | **4** | **5** | **6** | **7** | **8** | **9** | **10** | **NS NA**  **Strength** | |
| **d6 DOMESTIC LIFE** | **LEVEL OF FUNCTIONING** | | | | | | | | | | | | |
| **d620 Acquisition of goods and services**  Selecting, procuring and transporting all goods and services required for daily living, such as selecting, procuring, transporting and storing food, drink, clothing, cleaning materials, fuel, household items, utensils, cooking ware, play-material, domestic appliance and tools; procuring utilities and other household services. *Inclusions: shopping and gathering daily necessities.* | **0** | **1** | **2** | **3** | **4** | **5** | **6** | **7** | **8** | **9** | **10** | **NS NA**  **Strength** | |
| **d630 Preparing meals**  Planning, organizing, cooking and serving simple and complex meals for oneself and others, such as by making a menu, selecting edible food and drink, getting together ingredients for preparing meals, cooking with heat and preparing cold foods and drinks, and serving the food. *Inclusions: preparing simple and complex meals.* | **0** | **1** | **2** | **3** | **4** | **5** | **6** | **7** | **8** | **9** | **10** | **NS NA**  **Strength** | |
| **d640 Doing housework**  Managing a household by cleaning the house, washing clothes, using household appliances, storing food and disposing of garbage, such as by sweeping, mopping, washing counters, walls and other surfaces; collecting and disposing of household garbage; tidying rooms, closets and drawers; collecting, washing, drying, folding and ironing clothes; cleaning footwear; using brooms, brushes and vacuum cleaners; using washing machines, driers and irons. *Inclusions: washing and drying clothes and garments; cleaning cooking area and utensils; cleaning living area; using household appliances, storing daily necessities and disposing of garbage.* | **0** | **1** | **2** | **3** | **4** | **5** | **6** | **7** | **8** | **9** | **10** | **NS NA**  **Strength** | |
| **d660 Assisting others**  Assisting household members and others with their learning, communicating, self-care, movement, within the house or outside; being concerned about, or drawing other’s attention to, the well-being of household members and others. *Inclusions: assisting others with self-care, movement, communication, interpersonal relations, nutrition and health maintenance.* | **0** | **1** | **2** | **3** | **4** | **5** | **6** | **7** | **8** | **9** | **10** | **NS NA**  **Strength** | |
|  |  |  |  |  |  |  |  |  |  |  |  |  |  |


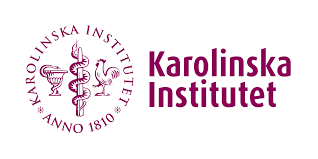

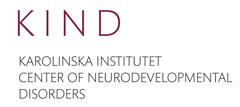


**0 = No difficulty/strength 10 = Complete difficulty/strength NS = Not specified NA = Not applicable**

| **d7 INTERPERSONAL INTERACTIONS AND RELATIONSHIPS** | **LEVEL OF FUNCTIONING** | | | | | | | | | | | |
| --- | --- | --- | --- | --- | --- | --- | --- | --- | --- | --- | --- | --- |
| **d710 Basic interpersonal interactions**  Interacting with people in a contextually and socially appropriate manner, such as by showing consideration and esteem when appropriate, or responding to the feelings of others. *Inclusions: showing respect, warmth, appreciation, and tolerance in relationships; responding to criticism and social cues in relationships; and using appropriate physical contact in relationships.* | **0** | **1** | **2** | **3** | **4** | **5** | **6** | **7** | **8** | **9** | **10** | **NS NA**  **Strength** |
| **d720 Complex interpersonal interactions**  Maintaining and managing interactions with other people, in a contextually and socially appropriate manner, such as by regulating emotions and impulses, controlling verbal and physical aggression, acting independently in social interactions, and acting in accordance with social rules and conventions. *Inclusions: playing with others, forming and terminating relationships; regulating behaviours within interactions; interacting according to social rules; and maintaining social space.* | **0** | **1** | **2** | **3** | **4** | **5** | **6** | **7** | **8** | **9** | **10** | **NS NA**  **Strength** |
| **d730 Relating with strangers**  Engaging in temporary contacts and links with strangers for specific purposes, such as when asking for information, directions or making a purchase. | **0** | **1** | **2** | **3** | **4** | **5** | **6** | **7** | **8** | **9** | **10** | **NS NA**  **Strength** |
| **d740 Formal relationships**  Creating and maintaining specific relationships in formal settings, such as with teachers, employers, professionals or service providers. *Inclusions: relating with persons in authority, with subordinates and with equals.* | **0** | **1** | **2** | **3** | **4** | **5** | **6** | **7** | **8** | **9** | **10** | **NS NA**  **Strength** |
| **d750 Informal social relationships**  Entering into relationships with others, such as casual relationships with people living in the same community or residence, or with co-workers, students, playmates or people with similar backgrounds or professions. *Inclusions: informal relationships with friends, neighbours, acquaintances, co-inhabitants and peers.* | **0** | **1** | **2** | **3** | **4** | **5** | **6** | **7** | **8** | **9** | **10** | **NS NA**  **Strength** |
| **d760 Family relationships**  Creating and maintaining kinship relationships, such as with members of the nuclear family, extended family, foster and adopted family and step-relationships, more distant relationships such as second cousins, or legal guardians. *Inclusions: parent-child and child-parent relationships, sibling and extended family relationships.* | **0** | **1** | **2** | **3** | **4** | **5** | **6** | **7** | **8** | **9** | **10** | **NS NA**  **Strength** |
| **d770 Intimate relationships**  Creating and maintaining close or romantic relationships between individuals, such as husband and wife, lovers or sexual partners. *Inclusions: romantic, spousal and sexual relationships.* | **0** | **1** | **2** | **3** | **4** | **5** | **6** | **7** | **8** | **9** | **10** | **NS NA**  **Strength** |


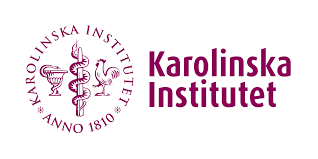

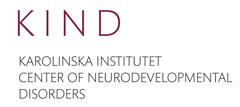


**0 = No difficulty/strength 10 = Complete difficulty/strength NS = Not specified NA = Not applicable**

| **d8 MAJOR LIFE AREAS** | **LEVEL OF FUNCTIONING** | | | | | | | | | | | |
| --- | --- | --- | --- | --- | --- | --- | --- | --- | --- | --- | --- | --- |
| **d810 Informal education**  Learning at home or in some other non-institutional setting, such as acquiring non-academic (e.g. crafts) or academic (e.g. home-schooling) skills from parents or family member in home or community. | **0** | **1** | **2** | **3** | **4** | **5** | **6** | **7** | **8** | **9** | **10** | **NS NA**  **Strength** |
| **d820 School education**  Gaining admission to school, education; engaging in all school-related responsibilities and privileges; learning the course material, subjects and other curriculum requirements in a primary or secondary education programme, including attending school regularly; working cooperatively with other students, taking direction from teachers, organizing, studying and completing assigned tasks and projects, and advancing to other stages of education. | **0** | **1** | **2** | **3** | **4** | **5** | **6** | **7** | **8** | **9** | **10** | **NS NA**  **Strength** |
| **d830 Higher education**  Engaging in the activities of advanced educational programmes in universities, colleges and professional schools and learning all aspects of the curriculum required for degrees, diplomas, certificates and other accreditations, such as completing a university bachelor’s or master’s course of study, medical school or other professional school. | **0** | **1** | **2** | **3** | **4** | **5** | **6** | **7** | **8** | **9** | **10** | **NS NA**  **Strength** |
| **d845 Acquiring, keeping and terminating a job**  Seeking, finding and choosing employment, being hired and accepting employment, maintaining and advancing through a job, trade, occupation or profession, and leaving a job in an appropriate manner. *Inclusions: seeking employment; preparing a resume or curriculum vitae; contacting employers and preparing interviews; maintaining a job; monitoring one’s own work performance; giving notice; and terminating a job.* | **0** | **1** | **2** | **3** | **4** | **5** | **6** | **7** | **8** | **9** | **10** | **NS NA**  **Strength** |
| **d850 Remunerative employment**  Engaging in all aspects of work, as an occupation, trade, profession or other form of employment, for payment, as an employee, full or part time, or self-employed, such as seeking employment and getting a job, doing the required tasks of the job, attending work on time as required, supervising other workers or being supervised, and performing required tasks alone or in groups. | **0** | **1** | **2** | **3** | **4** | **5** | **6** | **7** | **8** | **9** | **10** | **NS NA**  **Strength** |
| **d860 Basic economic transactions**  Engaging in any form of simple economic transaction, such as using money to purchase food or bartering, exchanging goods or services; or saving money. | **0** | **1** | **2** | **3** | **4** | **5** | **6** | **7** | **8** | **9** | **10** | **NS NA**  **Strength** |
| **d870 Economic self-sufficiency**  Having command over economic resources, from private or public sources, in order to ensure economic security for present and future needs. *Inclusions: personal economic resources and public economic entitlements.* | **0** | **1** | **2** | **3** | **4** | **5** | **6** | **7** | **8** | **9** | **10** | **NS NA**  **Strength** |


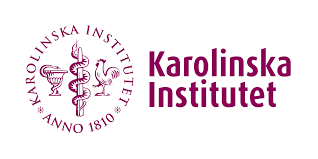

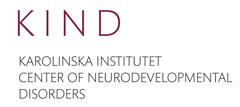


**0 = No difficulty/strength 10 = Complete difficulty/strength NS = Not specified NA = Not applicable**

| **d8 MAJOR LIFE AREAS** | **LEVEL OF FUNCTIONING** | | | | | | | | | | | |
| --- | --- | --- | --- | --- | --- | --- | --- | --- | --- | --- | --- | --- |
| **d880 Engagement in play**  Purposeful, sustained engagement in activities with objects, toys, materials or games, occupying oneself or with others. | **0** | **1** | **2** | **3** | **4** | **5** | **6** | **7** | **8** | **9** | **10** | **NS NA**  **Strength** |
| **d9 COMMUNITY, SOCIAL AND CIVIC LIFE** | **LEVEL OF FUNCTIONING** | | | | | | | | | | | |
| **d910 Community life**  Engaging in aspects of community social life, such as engaging in charitable organizations, service clubs or professional social organizations. *Inclusions: informal and formal associations; ceremonies.* | **0** | **1** | **2** | **3** | **4** | **5** | **6** | **7** | **8** | **9** | **10** | **NS NA**  **Strength** |
| **d920 Recreation and leisure**  Engaging in any form of play, recreational or leisure activity, such as informal or organized play and sports, programmes of physical fitness, relaxation, amusement or diversion, going to art galleries, museums, cinemas or theatres; engaging in crafts or hobbies, reading for enjoyment, playing musical instruments; sightseeing, tourism and travelling for pleasure. *Inclusions: games, sports, arts and culture, crafts, hobbies and socializing.* | **0** | **1** | **2** | **3** | **4** | **5** | **6** | **7** | **8** | **9** | **10** | **NS NA**  **Strength** |
| **d930 Religion and spirituality**  Engaging in religious or spiritual activities, organizations and practices for self-fulfillment, finding meaning, religious or spiritual value and establishing connection with a divine power, such as is involved in attending a church, temple, mosque or synagogue, praying or chanting for a religious purpose, and spiritual contemplation. *Inclusions: organized religion and spirituality.* | **0** | **1** | **2** | **3** | **4** | **5** | **6** | **7** | **8** | **9** | **10** | **NS NA**  **Strength** |
| **d940 Human rights**  Enjoying all nationally and internationally recognized rights that are accorded to people by virtue of their humanity alone, such as human rights as recognized by the United Nations Universal Declaration of Human Rights (1948) and the United Nations Standard Rules for the Equalization of Opportunities for Persons with Disabilities (1993); the United Nations Convention on the Rights of the Child (1989); the right to self-determination or autonomy; and the right to control over one’s destiny. | **0** | **1** | **2** | **3** | **4** | **5** | **6** | **7** | **8** | **9** | **10** | **NS NA**  **Strength** |
| **d950 Political life and citizenship**  Engaging in the social, political and governmental life of a citizen, having legal status as a citizen and enjoying the rights, protections, privileges and duties associated with that role, such as the right to vote and run for political office, to form political associations; enjoying the rights and freedoms associated with citizenship (e.g. the rights of freedom of speech, association, religion, protection against unreasonable search and seizure, the right to counsel, to a trial and other legal rights and protection against discrimination); having legal standing as a citizen. | **0** | **1** | **2** | **3** | **4** | **5** | **6** | **7** | **8** | **9** | **10** | **NS NA**  **Strength** |


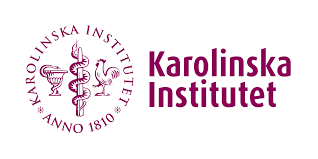

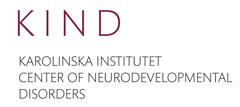


**0 = No difficulty/strength 10 = Complete difficulty/strength NS = Not specified NA = Not applicable**

| **ANY OTHER ACTIVITIES AND PARTICIPATION** | **LEVEL OF FUNCTIONING** | | | | | | | | | | | |
| --- | --- | --- | --- | --- | --- | --- | --- | --- | --- | --- | --- | --- |
|  | **0** | **1** | **2** | **3** | **4** | **5** | **6** | **7** | **8** | **9** | **10** | **NS NA**  **Strength** |
|  | **0** | **1** | **2** | **3** | **4** | **5** | **6** | **7** | **8** | **9** | **10** | **NS NA**  **Strength** |
|  | **0** | **1** | **2** | **3** | **4** | **5** | **6** | **7** | **8** | **9** | **10** | **NS NA**  **Strength** |
|  | **0** | **1** | **2** | **3** | **4** | **5** | **6** | **7** | **8** | **9** | **10** | **NS NA**  **Strength** |
|  | **0** | **1** | **2** | **3** | **4** | **5** | **6** | **7** | **8** | **9** | **10** | **NS NA**  **Strength** |
|  | **0** | **1** | **2** | **3** | **4** | **5** | **6** | **7** | **8** | **9** | **10** | **NS NA**  **Strength** |
|  | **0** | **1** | **2** | **3** | **4** | **5** | **6** | **7** | **8** | **9** | **10** | **NS NA**  **Strength** |
|  | **0** | **1** | **2** | **3** | **4** | **5** | **6** | **7** | **8** | **9** | **10** | **NS NA**  **Strength** |
|  | **0** | **1** | **2** | **3** | **4** | **5** | **6** | **7** | **8** | **9** | **10** | **NS NA**  **Strength** |
|  | **0** | **1** | **2** | **3** | **4** | **5** | **6** | **7** | **8** | **9** | **10** | **NS NA**  **Strength** |
|  | **0** | **1** | **2** | **3** | **4** | **5** | **6** | **7** | **8** | **9** | **10** | **NS NA**  **Strength** |
|  | **0** | **1** | **2** | **3** | **4** | **5** | **6** | **7** | **8** | **9** | **10** | **NS NA**  **Strength** |
|  | **0** | **1** | **2** | **3** | **4** | **5** | **6** | **7** | **8** | **9** | **10** | **NS NA**  **Strength** |
|  | **0** | **1** | **2** | **3** | **4** | **5** | **6** | **7** | **8** | **9** | **10** | **NS NA**  **Strength** |


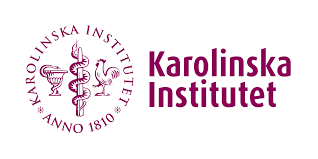

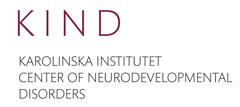


**Environmental factors component**

In this part you will rate categories related to environmental factors. Each category can EITHER be rated as barrier OR as facilitator. For the rating, it is important to use all available information in the medical records. Additional information is gathered through direct observation or interview with participant/caregiver. The information used for the rating can be up to three months old.

**Environmental factors** make up the physical, social and attitudinal environment in which people live and conduct their lives. The categories are to be rated from the perspective of the individual whose situation is being described. For example, kerb cuts without textured paving may be coded as a facilitator for a wheelchair user but as a barrier for a blind person.

**Facilitators** refer to issues such as the accessibility of a resource, and whether access is dependable or variable, of good or poor quality, and so on.

**Barriers** refer to hindering factors and whether the hindrance is great or small, or avoidable or not.

It should also be kept in mind that an environmental factor can be a barrier either because of its presence (for example negative attitudes towards people with disabilities) or its absence (for example, the unavailability of a needed service).

Facilitators are rated on a scale from 0 to +10, in which **0 stands for “Neither barrier/facilitator” and +10 for “Complete facilitator”.** When it comes to barriers, they are coded on a scale from 0 to -10, in which **0 stands for “Neither barrier/facilitator” and -10 for “Complete barrier”.**

The code **“Not specified” (NS)** is used in cases where there is not enough information to specify the level of barrier/facilitator. In other words, we know that an environmental factor is a barrier/facilitator but we don’t know the level of it.

The code **“Not applicable” (NA)** is used in cases where the specific code is not applicable to the participant, e.g. asking a question about family relationships when the participant does not have any family.


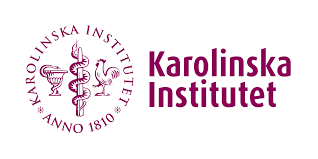

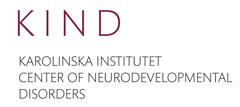


**0 = Neither barrier/facilitator -10 = Complete barrier +10 = Complete facilitator**

**NS = Not specified NA = Not applicable**

| **e1 PRODUCTS AND TECHNOLOGY** | **LEVEL OF BARRIER/FACILITATOR** | | | | | | | | | | | | |
| --- | --- | --- | --- | --- | --- | --- | --- | --- | --- | --- | --- | --- | --- |
| **e110 Products or substances for personal consumption**  Any natural or human-made object or substance gathered, processed or manufactured for ingestion. *Inclusions: food (including breast milk), drink and drugs.* |  | **-1** | **-2** | **-3** | **-4** | **-5** | **-6** | **-7** | **-8** | **-9** | **-10** |  |  |
|  | **0** |  |  |  |  |  |  |  |  |  |  | **NS** | **NA** |
|  |  | **+1** | **+2** | **+3** | **+4** | **+5** | **+6** | **+7** | **+8** | **+9** | **+10** |  |  |
| **e115 Products and technology for personal use in daily living**  Equipment, products and technologies used by people in daily activities, including those adapted or specially designed, located in, on or near the person using them. |  | **-1** | **-2** | **-3** | **-4** | **-5** | **-6** | **-7** | **-8** | **-9** | **-10** |  |  |
|  | **0** |  |  |  |  |  |  |  |  |  |  | **NS** | **NA** |
|  |  | **+1** | **+2** | **+3** | **+4** | **+5** | **+6** | **+7** | **+8** | **+9** | **+10** |  |  |
| **e120 Products and technology for personal indoor and outdoor mobility and transportation**  Equipment, products and technologies used by people in activities of moving inside and outside buildings, including those adapted or specially designed, located in, on or near the person using them. | **-1 -2 -3 -4 -5 -6 -7 -8 -9 -10**  **0 NS NA**  **+1 +2 +3 +4 +5 +6 +7 +8 +9 +10** | | | | | | | | | | | | |
| **e125 Products and technology for communication**  Equipment, products and technologies used by people in activities of sending and receiving information, such as optical and auditory devices, audio recorders and receivers, television and video equipment, telephone devices, sound transmission systems and face-to-face communication devices, not adapted or specially designed. | **-1 -2 -3 -4 -5 -6 -7 -8 -9 -10**  **0 NS NA**  **+1 +2 +3 +4 +5 +6 +7 +8 +9 +10** | | | | | | | | | | | | |
| **e150 Design, construction and building products and technology of buildings for public use**  Products and technology that constitute an individual’s indoor and outdoor human-made environment that is planned, designed and constructed for public use, including those adapted or specially designed. *Inclusions: design, construction and building products and technology of entrances and exits, facilities and routing.* | **-1 -2 -3 -4 -5 -6 -7 -8 -9 -10**  **0 NS NA**  **+1 +2 +3 +4 +5 +6 +7 +8 +9 +10** | | | | | | | | | | | | |


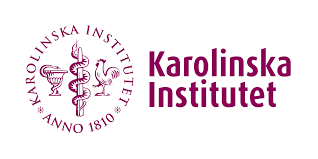

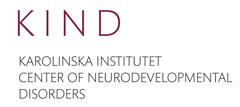


**0 = Neither barrier/facilitator -10 = Complete barrier +10 = Complete facilitator**

**NS = Not specified NA = Not applicable**

| **e1 PRODUCTS AND TECHNOLOGY** | **LEVEL OF BARRIER/FACILITATOR** | | | | | | | | | | | | |
| --- | --- | --- | --- | --- | --- | --- | --- | --- | --- | --- | --- | --- | --- |
| **e155 Design, construction and building products and technology of buildings for private use**  Products and technology that constitute an individual’s indoor and outdoor human-made environment that is planned, designed and constructed for private use (e.g. home, dwelling), including those adapted or specially designed. *Inclusions: design, construction and building products and technology of entrances and exits, facilities and routing.* |  | **-1** | **-2** | **-3** | **-4** | **-5** | **-6** | **-7** | **-8** | **-9** | **-10** |  |  |
|  | **0** |  |  |  |  |  |  |  |  |  |  | **NS** | **NA** |
|  |  | **+1** | **+2** | **+3** | **+4** | **+5** | **+6** | **+7** | **+8** | **+9** | **+10** |  |  |
| **e165 Assets**  Products or objects of economic exchange such as money, goods, property and other valuables that an individual owns or which he or she has rights of use or rights of benefit, such as child support payment or wills for children or dependent persons. *Inclusions: tangible and intangible products and goods, financial assets.* |  | **-1** | **-2** | **-3** | **-4** | **-5** | **-6** | **-7** | **-8** | **-9** | **-10** |  |  |
|  | **0** |  |  |  |  |  |  |  |  |  |  | **NS** | **NA** |
|  |  | **+1** | **+2** | **+3** | **+4** | **+5** | **+6** | **+7** | **+8** | **+9** | **+10** |  |  |
| **e2 NATURAL ENVIRONMENT AND HUMAN-MADE CHANGES TO ENVIRONMENT** | **LEVEL OF BARRIER/FACILITATOR** | | | | | | | | | | | | |
| **e225 Climate**  Meteorological features and events, such as the weather. *Inclusions: temperature, humidity, atmospheric pressure, precipitation, wind and seasonal variations.* | **-1 -2 -3 -4 -5 -6 -7 -8 -9 -10**  **0 NS NA**  **+1 +2 +3 +4 +5 +6 +7 +8 +9 +10** | | | | | | | | | | | | |
| **e240 Light**  Electromagnetic radiation by which things are made visible by either sunlight or artificial lighting (e.g. candles, oil or paraffin lamps, fires and electricity), and which may provide useful or distracting information about the world. *Inclusions: light intensity; light quality; colour contrasts.* | **-1 -2 -3 -4 -5 -6 -7 -8 -9 -10**  **0 NS NA**  **+1 +2 +3 +4 +5 +6 +7 +8 +9 +10** | | | | | | | | | | | | |
| **e250 Sound**  A phenomenon that is, or may be heard, such as banging, ringing, thumping, singing, whistling, yelling or buzzing, in any volume, timbre or tone, and that may provide useful or distracting information about the world. *Inclusions: sound intensity; sound quality.* | **-1 -2 -3 -4 -5 -6 -7 -8 -9 -10**  **0 NS NA**  **+1 +2 +3 +4 +5 +6 +7 +8 +9 +10** | | | | | | | | | | | | |


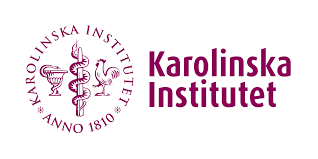

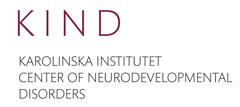


**0 = Neither barrier/facilitator -10 = Complete barrier +10 = Complete facilitator**

**NS = Not specified NA = Not applicable**

| **e3 SUPPORT AND RELATIONSHIPS** | **LEVEL OF BARRIER/FACILITATOR** | | | | | | | | | | | | |
| --- | --- | --- | --- | --- | --- | --- | --- | --- | --- | --- | --- | --- | --- |
| **e310 Immediate family**  Individuals related by birth, marriage or other relationship recognized by the culture as immediate family, such as spouses, partners, parents, siblings, children, foster parents, adoptive parents and grandparents. |  | **-1** | **-2** | **-3** | **-4** | **-5** | **-6** | **-7** | **-8** | **-9** | **-10** |  |  |
|  | **0** |  |  |  |  |  |  |  |  |  |  | **NS** | **NA** |
|  |  | **+1** | **+2** | **+3** | **+4** | **+5** | **+6** | **+7** | **+8** | **+9** | **+10** |  |  |
| **e315 Extended family**  Individuals related through family or marriage, or other relationships recognized by the culture as extended family, such as aunts, uncles, nephews and nieces. | **-1 -2 -3 -4 -5 -6 -7 -8 -9 -10**  **0 NS NA**  **+1 +2 +3 +4 +5 +6 +7 +8 +9 +10** | | | | | | | | | | | | |
| **e320 Friends**  Individuals who are close and ongoing participants in relationships characterized by trust and mutual support. | **-1 -2 -3 -4 -5 -6 -7 -8 -9 -10**  **0 NS NA**  **+1 +2 +3 +4 +5 +6 +7 +8 +9 +10** | | | | | | | | | | | | |
| **e325 Acquaintances, peers, colleagues, neighbours and community members**  Individuals who are familiar to each other as acquaintances, peers, colleagues, neighbours, and community members, in situations of work, school, recreation, or other aspects of life, and who share demographic features such as age, gender, religious creed or ethnicity or pursue common interests. | **-1 -2 -3 -4 -5 -6 -7 -8 -9 -10**  **0 NS NA**  **+1 +2 +3 +4 +5 +6 +7 +8 +9 +10** | | | | | | | | | | | | |
| **e330 People in positions of authority**  Individuals who have decision-making responsibilities for others and who have socially defined influence or power based on their social, economic, cultural or religious roles in society, such as teachers, employers, supervisors, religious leaders, substitute decision-makers, guardians or trustees. | **-1 -2 -3 -4 -5 -6 -7 -8 -9 -10**  **0 NS NA**  **+1 +2 +3 +4 +5 +6 +7 +8 +9 +10** | | | | | | | | | | | | |
| **e340 Personal care providers and personal assistants**  Individuals who provide services as required to support individuals in their daily activities and maintenance of performance at work, education or other life situation, provided either through public or private funds, or else on a voluntary basis, such as providers of support for home-making and maintenance, personal assistants, transport assistants, paid help, nannies and others who function as primary caregivers. | **-1 -2 -3 -4 -5 -6 -7 -8 -9 -10**  **0 NS NA**  **+1 +2 +3 +4 +5 +6 +7 +8 +9 +10** | | | | | | | | | | | | |


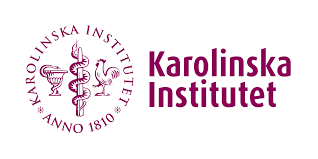

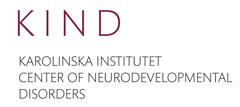


**0 = Neither barrier/facilitator -10 = Complete barrier +10 = Complete facilitator**

**NS = Not specified NA = Not applicable**

| **e3 SUPPORT AND RELATIONSHIPS** | **LEVEL OF BARRIER/FACILITATOR** | | | | | | | | | | | | |
| --- | --- | --- | --- | --- | --- | --- | --- | --- | --- | --- | --- | --- | --- |
| **e355 Health professionals**  All service providers working within the context of the health system, such as doctors, nurses, physiotherapists, occupational therapists, speech therapists, audiologists, orthoist-prosthetists, medical social workers. |  | **-1** | **-2** | **-3** | **-4** | **-5** | **-6** | **-7** | **-8** | **-9** | **-10** |  |  |
|  | **0** |  |  |  |  |  |  |  |  |  |  | **NS** | **NA** |
|  |  | **+1** | **+2** | **+3** | **+4** | **+5** | **+6** | **+7** | **+8** | **+9** | **+10** |  |  |
| **e360 Other professionals**  All service providers working outside the health system, including social workers, lawyers, teachers, architects, and designers. | **-1 -2 -3 -4 -5 -6 -7 -8 -9 -10**  **0 NS NA**  **+1 +2 +3 +4 +5 +6 +7 +8 +9 +10** | | | | | | | | | | | | |
| **e4 ATTITUDES** | **LEVEL OF BARRIER/FACILITATOR** | | | | | | | | | | | | |
| **e410 Individual attitudes of immediate family members**  General or specific opinions and beliefs of immediate family members about the person or about other matters (e.g. social, political and economic issues), that influence individual behavior and actions. | **-1 -2 -3 -4 -5 -6 -7 -8 -9 -10**  **0 NS NA**  **+1 +2 +3 +4 +5 +6 +7 +8 +9 +10** | | | | | | | | | | | | |
| **e420 Individual attitudes of friends**  General or specific opinions and beliefs of friends about the person or about other matters (e.g. social, political and economic issues), that influence individual behavior and actions. | **-1 -2 -3 -4 -5 -6 -7 -8 -9 -10**  **0 NS NA**  **+1 +2 +3 +4 +5 +6 +7 +8 +9 +10** | | | | | | | | | | | | |
| **e425 Individual attitudes of acquaintances, peers, colleagues, neighbours and community members**  General or specific opinions and beliefs of acquaintances, peers, colleagues, neighbours and community members about the person or about other matters (e.g. social, political and economic issues), that influence individual behavior and actions. | **-1 -2 -3 -4 -5 -6 -7 -8 -9 -10**  **0 NS NA**  **+1 +2 +3 +4 +5 +6 +7 +8 +9 +10** | | | | | | | | | | | | |
| **e440 Individual attitudes of personal care providers and personal assistants**  General or specific opinions and beliefs of personal care providers and personal assistants about the person or about other matters (e.g. social, political and economic issues), that influence individual behavior and actions. | **-1 -2 -3 -4 -5 -6 -7 -8 -9 -10**  **0 NS NA**  **+1 +2 +3 +4 +5 +6 +7 +8 +9 +10** | | | | | | | | | | | | |
| **e450 Individual attitudes of health professionals**  General or specific opinions and beliefs of health professionals about the person or about other matters (e.g. social, political and economic issues), that influence individual behavior and actions. | **-1 -2 -3 -4 -5 -6 -7 -8 -9 -10**  **0 NS NA**  **+1 +2 +3 +4 +5 +6 +7 +8 +9 +10** | | | | | | | | | | | | |


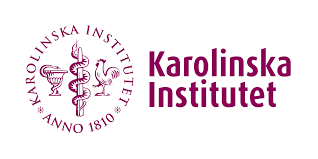

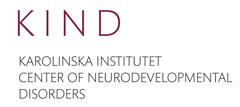


**0 = Neither barrier/facilitator -10 = Complete barrier +10 = Complete facilitator**

**NS = Not specified NA = Not applicable**

| **e4 ATTITUDES** | **LEVEL OF BARRIER/FACILITATOR** |
| --- | --- |
| **e455 Individual attitudes of other professionals**  General or specific opinions and beliefs of health-related and other professionals about the person or about other matters (e.g. social, political and economic issues), that influence individual behavior and actions. | **-1 -2 -3 -4 -5 -6 -7 -8 -9 -10**  **0 NS NA**  **+1 +2 +3 +4 +5 +6 +7 +8 +9 +10** |
| **e460 Societal attitudes**  General or specific opinions and beliefs generally held by people of a culture, society, subcultural or other social group about other individuals or about other social, political and economic issues, that influence group or individual behavior and actions. | **-1 -2 -3 -4 -5 -6 -7 -8 -9 -10**  **0 NS NA**  **+1 +2 +3 +4 +5 +6 +7 +8 +9 +10** |
| **e465 Social norms, practices and ideologies**  Customs, practices, rules and abstract systems of values and normative beliefs (e.g. ideologies, normative world views and moral philosophies) that arise within social contexts and that affect or create societal and individual practices and behaviours, such as social norms of moral and religious behavior or etiquette; religious doctrine and resulting norms and practices; norms governing rituals or social gatherings. | **-1 -2 -3 -4 -5 -6 -7 -8 -9 -10**  **0 NS NA**  **+1 +2 +3 +4 +5 +6 +7 +8 +9 +10** |
| **e5 SERVICES, SYSTEMS AND POLICIES** | **LEVEL OF BARRIER/FACILITATOR** |
| **e525 Housing services, systems and policies**  Services, systems and policies for the provision of shelters, dwellings or lodging for people. | **-1 -2 -3 -4 -5 -6 -7 -8 -9 -10**  **0 NS NA**  **+1 +2 +3 +4 +5 +6 +7 +8 +9 +10** |
| **e535 Communication services, systems and policies**  Services, systems and policies for the transmission and exchange of information. | **-1 -2 -3 -4 -5 -6 -7 -8 -9 -10**  **0 NS NA**  **+1 +2 +3 +4 +5 +6 +7 +8 +9 +10** |
| **e540 Transportation services, systems and policies**  Services, systems and policies for enabling people or goods to move or be moved from one location to another. | **-1 -2 -3 -4 -5 -6 -7 -8 -9 -10**  **0 NS NA**  **+1 +2 +3 +4 +5 +6 +7 +8 +9 +10** |
| **e550 Legal services, systems and policies**  Services, systems and policies concerning the legislation and other law of a country. | **-1 -2 -3 -4 -5 -6 -7 -8 -9 -10**  **0 NS NA**  **+1 +2 +3 +4 +5 +6 +7 +8 +9 +10** |
| **e570 Social security services, systems and policies**  Services, systems and policies aimed at providing income support to people who, because of age, poverty, unemployment, health condition or disability, require public assistance that is funded either by general tax revenues or contributory schemes. | **-1 -2 -3 -4 -5 -6 -7 -8 -9 -10**  **0 NS NA**  **+1 +2 +3 +4 +5 +6 +7 +8 +9 +10** |


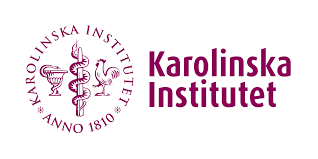

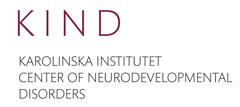


**0 = Neither barrier/facilitator -10 = Complete barrier +10 = Complete facilitator**

**NS = Not specified NA = Not applicable**

| **e5 SERVICES, SYSTEMS AND POLICIES** | **LEVEL OF BARRIER/FACILITATOR** |
| --- | --- |
| **e575 General social support services, systems and policies**  Services, systems and policies aimed at providing support to those requiring assistance in areas such as shopping, housework, transport, child care, respite care, self-care and care of others, in order to function more fully in society. | **-1 -2 -3 -4 -5 -6 -7 -8 -9 -10**  **0 NS NA**  **+1 +2 +3 +4 +5 +6 +7 +8 +9 +10** |
| **e580 Health services, systems and policies**  Services, systems and policies for preventing and treating health problems, providing medical rehabilitation and promoting a healthy lifestyle. | **-1 -2 -3 -4 -5 -6 -7 -8 -9 -10**  **0 NS NA**  **+1 +2 +3 +4 +5 +6 +7 +8 +9 +10** |
| **e585 Education and training services, systems and policies**  Services, systems and policies for the acquisition, maintenance and improvement of knowledge, expertise and vocational or artistic skills. See UNESCO’s International Standard Classification of Education (ISCED-1997). | **-1 -2 -3 -4 -5 -6 -7 -8 -9 -10**  **0 NS NA**  **+1 +2 +3 +4 +5 +6 +7 +8 +9 +10** |
| **e590 Labour and employment services, systems and policies**  Services, systems and policies related to finding suitable work for persons who are unemployed or looking for different work, or to support individuals already employed who are seeking promotion. | **-1 -2 -3 -4 -5 -6 -7 -8 -9 -10**  **0 NS NA**  **+1 +2 +3 +4 +5 +6 +7 +8 +9 +10** |


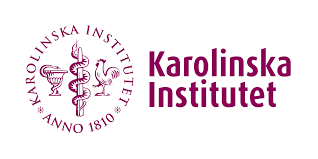

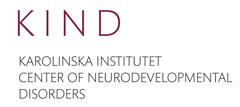


**0 = Neither barrier/facilitator -10 = Complete barrier +10 = Complete facilitator**

**NS = Not specified NA = Not applicable**

| **ANY OTHER ENVIRONMENTAL FACTORS** | **LEVEL OF BARRIER/FACILITATOR** |
| --- | --- |
|  | **-1 -2 -3 -4 -5 -6 -7 -8 -9 -10**  **0 NS NA**  **+1 +2 +3 +4 +5 +6 +7 +8 +9 +10** |
|  | **-1 -2 -3 -4 -5 -6 -7 -8 -9 -10**  **0 NS NA**  **+1 +2 +3 +4 +5 +6 +7 +8 +9 +10** |
|  | **-1 -2 -3 -4 -5 -6 -7 -8 -9 -10**  **0 NS NA**  **+1 +2 +3 +4 +5 +6 +7 +8 +9 +10** |
|  | **-1 -2 -3 -4 -5 -6 -7 -8 -9 -10**  **0 NS NA**  **+1 +2 +3 +4 +5 +6 +7 +8 +9 +10** |
|  | **-1 -2 -3 -4 -5 -6 -7 -8 -9 -10**  **0 NS NA**  **+1 +2 +3 +4 +5 +6 +7 +8 +9 +10** |
|  | **-1 -2 -3 -4 -5 -6 -7 -8 -9 -10**  **0 NS NA**  **+1 +2 +3 +4 +5 +6 +7 +8 +9 +10** |
|  | **-1 -2 -3 -4 -5 -6 -7 -8 -9 -10**  **0 NS NA**  **+1 +2 +3 +4 +5 +6 +7 +8 +9 +10** |
|  | **-1 -2 -3 -4 -5 -6 -7 -8 -9 -10**  **0 NS NA**  **+1 +2 +3 +4 +5 +6 +7 +8 +9 +10** |
|  | **-1 -2 -3 -4 -5 -6 -7 -8 -9 -10**  **0 NS NA**  **+1 +2 +3 +4 +5 +6 +7 +8 +9 +10** |
|  | **-1 -2 -3 -4 -5 -6 -7 -8 -9 -10**  **0 NS NA**  **+1 +2 +3 +4 +5 +6 +7 +8 +9 +10** |


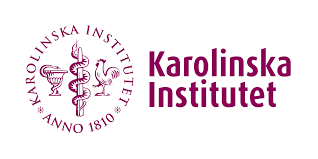

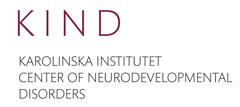


**Part 4: Personal factors**

| ***Here you can include Personal factors that hamper or support the individual’s functioning level (e.g. lifestyle, habits, social background, education, life events, race/ethnicity, sexual orientation and assets of the individual).*** |
| --- |
|  |
